# Evaluation of the e‐Surveyor Mobile Application for Undertaking Plant Surveys and Predicting Habitat Type

**DOI:** 10.1002/ece3.73901

**Published:** 2026-06-29

**Authors:** Lucy E. Ridding, Morag McCracken, Zephyr Orsler, Emily V. Upcott, Nadine Mitschunas, Karolis Kazlauskis, Zeke Marshall, Grace Skinner, Simon M. Smart, Colin A. Harrower, Oliver L. Pescott, Richard F. Pywell, Tom A. August

**Affiliations:** ^1^ UK Centre for Ecology and Hydrology Wallingford UK; ^2^ Flumens Kaunas Lithuania; ^3^ UK Centre for Ecology and Hydrology Bailrigg UK

**Keywords:** automated identification, broad habitat, citizen science, habitat classification, mobile application

## Abstract

Mobile applications with automated species identification can assist citizen scientists in undertaking plant and habitat surveys. Whilst a high level of accuracy for these applications has been reported, very little testing has been done with citizen scientists in the field. If such applications are going to be used to support biodiversity research and conservation management, they need to be sufficiently accurate and functional for their intended use. We evaluated the accuracy of the e‐Surveyor mobile application, which includes automated identification for plant species and habitat prediction. We compared species lists and derived habitat associations collected by citizen scientists using the application in the field, with data recorded by expert botanists within the same survey plots. We also assessed the user experience via a questionnaire. Thirty‐seven citizen scientists attended the e‐Surveyor workshops and completed a questionnaire, with 51 individual plant surveys submitted across the three habitat types: calcareous grassland, neutral grassland and improved grassland. On average, experts recorded more plant species per plot compared with citizen scientists. Of the species recorded by citizen scientists on e‐Surveyor that were known to be present, 71% were correctly identified to species level, though typically only 45% of all observable species in the plot according to the expert botanists were captured correctly by the citizen scientists. Eighty per cent of surveys identified the correct first broad habitat and 25% identified the correct first phytosociological community suggested by the application. Citizen scientists provided valuable input through the questionnaire, including improvements to e‐Surveyor and future use cases. Most citizen scientists were able to accurately identify almost half of the observable plant species present and determine the correct broad habitat using e‐Surveyor, regardless of their botanical skill level. This suggests that the application is a useful tool for supporting biological recording, whilst improving confidence, knowledge and engagement with nature.

## Graphical abstract

We evaluated the accuracy of the e‐Surveyor mobile application, which includes automated identification for plant species and habitat prediction. We compared species lists collected by citizen scientists using the application in the field with data recorded by expert botanists. Most citizen scientists were able to accurately identify almost half of the observable plant species present and determine the correct broad habitat using e‐Surveyor, regardless of their botanical skill level. This suggests that the application is a useful tool for supporting biological recording, whilst improving confidence, knowledge and engagement with nature.

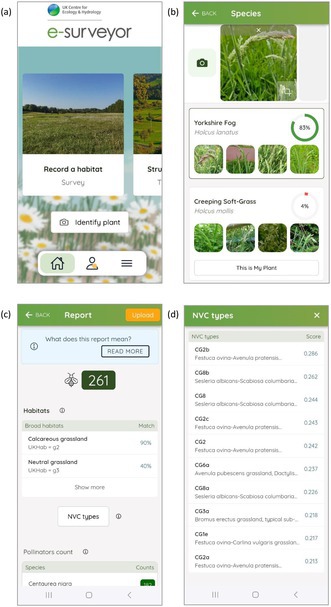

## 1 Introduction

Good knowledge of the species and habitats present on a site is fundamental to their effective management and conservation. However, traditional biodiversity monitoring is labour‐intensive and requires trained experts, making it expensive and impractical to undertake over large geographic regions in a consistent way. Citizen science is becoming an increasingly effective tool for collecting biodiversity data (Pocock et al. 2017), as well as playing an important role in educating, sharing knowledge and improving well‐being (McKinley et al. 2017; Pocock et al. 2023). The key challenges of citizen science for vegetation and habitat surveys are the high level of skill required to correctly identify the plant species present and the ability to classify plant communities and how these form habitats (Poore 1955). Over the last decade, the development of new automated species identification systems and tools which can assist citizen scientists in undertaking biological recording has accelerated, which may help to address some of these challenges.

Automated species identification systems are now readily available through mobile applications, following significant advances in machine learning algorithms, including deep learning methods such as convolutional neural networks (CNNs) (LeCun et al. 2015). Deep learning methods have also been used to assign vegetation‐plot records to their respective habitat types (Leblanc et al. 2024). The current landscape of biodiversity recording apps is diverse, including both free and subscription‐based platforms, as well as applications that contribute observations to open research infrastructures and those that retain data within proprietary systems (Truong and Van der Wal 2024). Several generalist image identification applications such as Google Lens are publicly available, as well as those specifically developed to record plants including Flora Incognita (Mäder et al. 2021) and Pl@ntNet (Affouard et al. 2017). Interest in these applications has increased rapidly in recent years (Google Trends 2025), which offer new opportunities for members of the public to engage with nature. However, for such tools to be useful for collecting data to support biodiversity research and conservation management, the automated species identification and associated outputs need to be sufficiently accurate and structured for their intended use.

Several studies have tested and compared plant identification systems against human experts. The LifeCLEF international framework brings together international research groups to compare machine learning and human experts. The latest assessment within the plant identification task used the largest published plant dataset, totalling 4 million images across 80,000 species, which included both trusted labelled data and ‘noisy’ web data (e.g., wrong species labels, drawings or herbarium sheets). They found the performance of the deep learning models was now close to human expertise (Goëau et al. 2023). Other studies have tested existing applications, for example, August et al. (2020) applied the Pl@ntNet classifier to social media images, whilst Hart et al. (2023) assessed five plant identification applications using professionally identified images. Whilst these studies confirm a high level of accuracy for automated plant species identification, few have evaluated such applications using citizen scientists in the field. Instead, photos tend to be extracted from existing databases (Bilyk et al. 2020; Jones 2020; Kress et al. 2018; Mäder et al. 2021) or are taken by professional ecologists (Hart et al. 2023). One exception, undertaken by Pärtel et al. (2021), investigated plant image identification accuracy, from both database images and under field conditions using the Flora Incognita application. They found 79.6% of species were correctly identified from the database images, whilst accuracy using the application in the field reached 85.3%, however this was not tested with citizen scientists.

Evaluating automated identification applications in the field with citizen scientists is not only important for testing the accuracy of the identification algorithms themselves, but also for understanding what plant species are likely to be detected and photographed (Chen et al. 2009; Groom and Whild 2017; McCarthy et al. 2013). For instance, people are usually attracted to plants with larger flowers and vibrant colours (Hůla and Flegr 2016; Marcenò et al. 2021). Understanding which species are captured by users is important for determining any biases, which can influence the accuracy of derived information, such as plant distributions and communities, and consequently habitat types. Characterising habitat types can be challenging in the UK, since several different habitat classification systems exist, which employ different methodologies. For example, the UK broad habitat system is physiognomic (JNCC 2019), similar to the UK Habitat Classification (UKHab Ltd. 2023), whilst the British National Vegetation Classification (NVC) (Rodwell 1998) is phytosociological. Furthermore, some habitat types, such as species‐rich grasslands, can be very complex due to the number of species present, and therefore a good understanding of the dominant species in these habitats/communities is important. Thus, the extent to which citizen scientists can use automated identification applications, to firstly identify species correctly and secondly derive habitat type, is important for understanding whether these systems can be used for biological recording to aid biodiversity conservation and research. Furthermore, testing with citizen scientists in the field is important for gauging user experience and understanding how this technology may be used, yet currently there is little information on this (Hart et al. 2023). Understanding whether citizen scientists enjoy using these systems and the features they find most useful is important for determining future usage and to identify where improvements to usability and knowledge transfer can be integrated. Understanding which biological recording activities this technology could assist with, can help to target improvements into existing applications or the design of new tools.

In this study we evaluate the accuracy of the e‐Surveyor mobile application, which includes automated identification for vascular plant species (hereafter plants) and habitat prediction. We compare species lists and derived habitat associations collected by citizen scientists using the application in the field with data recorded by expert botanists within the same survey plots. Furthermore, we assess the user experience via a questionnaire to understand application performance and potential future uses. We address the following research questions:

- Do citizen scientists record fewer species than expert botanists during a survey?
- Are citizen scientists able to accurately record the species they detect using the application?
- Does the plant type, flowering status and species abundance influence whether the species is more likely to be captured accurately?
- Does the application derive the correct habitat type based on the species list recorded by the citizen scientist and is this influenced by the number of correct species captured?
- Are there any patterns between a citizen scientist's' confidence/knowledge and their ability to accurately record plant lists and derive correct habitat classifications using the e‐Surveyor application?
- How do citizen scientists find the experience of using e‐Surveyor?

## 2 Materials and Methods

### 2.1 e‐Surveyor Mobile Application

**FIGURE 1:**
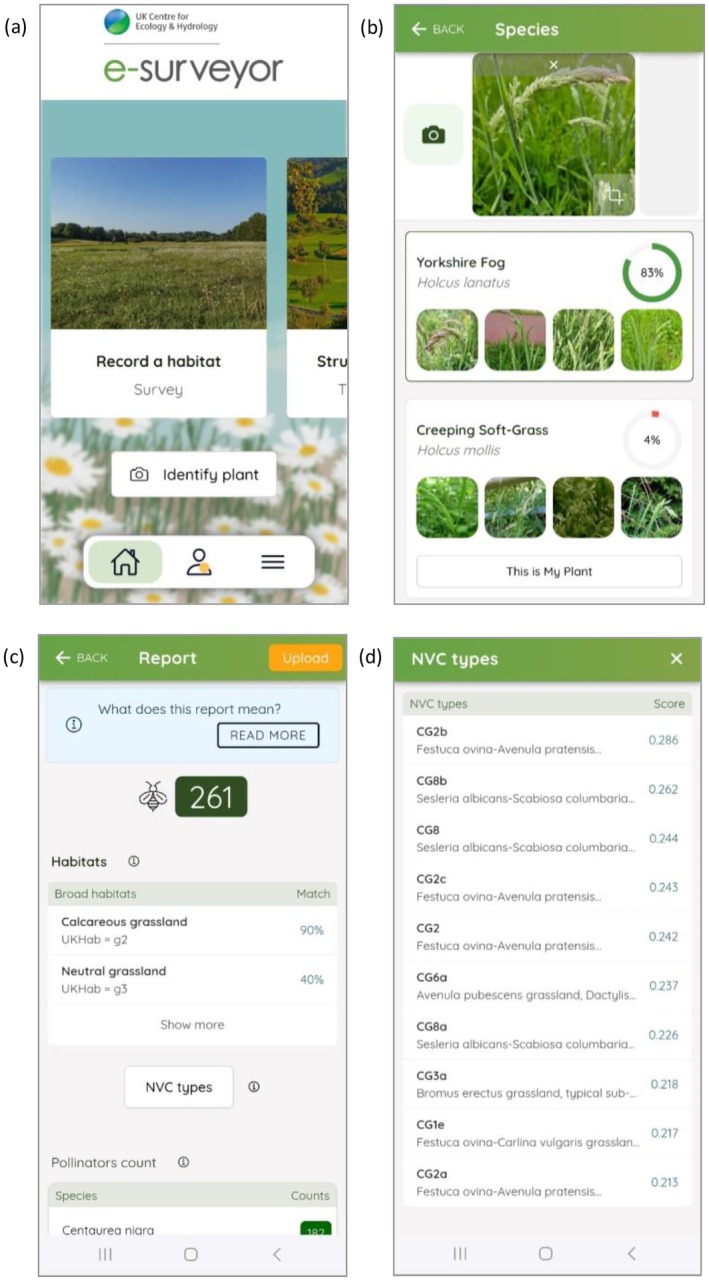
Screenshots from the e‐Surveyor application including (a) the landing page showing the ‘Record a habitat’ survey function, (b) species suggestions using the AI technology and (c) the broad habitat report provided to users following the successful completion of a ‘Record a habitat’ survey and (d) the National Vegetation Classification (NVC) report.

e‐Surveyor is a free mobile application developed by the UK Centre for Ecology & Hydrology; it allows farmers, landowners and citizen scientists to assess the quality of the habitats they manage and have an interest in conserving (https://esurveyor.ceh.ac.uk/). In this study we tested the habitat survey function (Figure 1a), which allows citizen scientists to generate a plant species list by manually entering known plant species or by using the in‐built AI technology to generate species suggestions, which are provided by the Pl@ntNet API via the non‐profit access route (Affouard et al. 2017, https://my.plantnet.org/); the API uses Kew Royal Botanical Gardens' Plants Of The World Online (POWO) checklist as the taxonomic backbone. We use v2 of the API, specifically the ‘identify’ end point with ‘weurope’ specified as the project to ensure only species found in Western Europe are returned (for details see: https://my‐api.plantnet.org/#/my‐api/postV2IdentifyProject). For each species suggestion Pl@ntNet provides a confidence score (percentage) (Figure 1b). One photo is required, however up to five photos can be added per individual plant, which may increase the confidence score. The species with the highest confidence score is automatically selected by the application, however users can override this selection if they disagree with the suggestion. Identification requires internet access. When this is not available the photos are left unclassified until an internet connection is restablished, at which point they are submitted to the Pl@ntNet API for identification.

Following the completion of the survey users are presented with a report, which details the associated habitat types from three classification systems: UK broad habitat (JNCC 2019), UK Habitat Classification (UKHab Ltd. 2023) and British National Vegetation Classification (NVC) (Rodwell 1998) (Figure 1c). The broad habitat assignment is based on the habitat preferences of Hill et al. (2004) for each individual vascular plant species recorded in the survey. The habitat match score is calculated as the proportion of species present in the survey which have a preference for the respective habitat, noting that some species will only be associated with one habitat whilst others may be associated with multiple (Figure 1c). UKHab codes correspond with the UK broad habitat classification system (JNCC 2019) and are presented below the broad habitats on the application (Figure 1c). The NVC output requires the user to have recorded a full exhaustive survey (i.e., to record all species present in the survey area) before displaying the top ten likely associated NVC communities with a similarity index (Jaccard) for each (Figure 1d). This output is derived using the NVC pseudo‐quadrat methodology described in Tipping et al. (2017). This method uses a Bernoulli trial to determine the presence or absence of each species, with the probability of a successful trial equal to the relative frequency of occurrence of each species in a NVC community. Unlike the broad habitat matching, the in‐application NVC classification can use bryophytes, charophytes, macroalgae and macrolichens, in addition to vascular plants.

### 2.2 Field Workshops

To test the habitat functionality in the e‐Surveyor application we ran seven field workshops with different citizen science groups ranging from 4 to 10 participants in size. Citizen scientists (termed ‘participants’ from hereon) were those with an interest in biological recording, wildlife applications or were members of a practical conservation group. Workshops took place in central and southern England between June and August 2024 to coincide with the peak flowering period in England, since flowers are a key identifying feature and are generally easy to photograph using a camera phone. Workshops were held in three different habitat types ranging from high to low complexity based on the expected number of species present: UKHab v2.01 broad habitat types of calcareous grassland (g2; ‘vegetation dominated by grasses and herbs on shallow, well‐drained soils that are rich in bases (principally calcium carbonate) formed by the weathering of chalk and other types of limestone or base‐rich rock’), neutral grassland (g3; ‘vegetation dominated by grasses and herbs on a range of neutral soils, usually with a pH of 4.5‐5.5’) and modified grassland (hereon ‘improved grassland’, g4; ‘dominated by a few fast‐growing grasses on fertile, neutral soils’); all definitions taken from UKHab Ltd. (2023).

At each workshop, participants were introduced to e‐Surveyor before being asked to undertake a ‘Record a habitat’ survey on the application. We instructed participants to record all vascular plants within a 25 m^2^ plot. This plot size was selected for this study to ensure participants had sufficient vegetation of interest to explore, particularly in the lower complexity habitats. Furthermore, this plot size is also consistent with plot sizes used in other citizen science recording schemes, for example, the National Plant Monitoring Scheme (NPMS) (Pescott et al. 2019). Participants were able to utilise the camera function with the inbuilt AI technology to generate species suggestions, otherwise they had the option to record a plant species manually if they were confident with the identification. Participants were able to take up to five photos per individual plant and could override species suggestions thought to be incorrect. Each participant recorded their own 25 m^2^ plot independently, though a small number of plots were recorded by two participants (*n* = 5).

Each plot recorded by a participant was also surveyed by 2–3 professional expert botanists (authors LER, EVU, NM) (termed ‘experts’ from hereon) that worked independently to ensure an accurate full inventory list was collated. Only a small number of plots were surveyed by one expert, predominantly in the simplest habitat (improved grassland). Experts recorded a simple abundance using the DAFOR scale (Dominant, Abundant, Frequent, Occasional or Rare) for each vascular plant present, and recorded whether it was flowering. We defined flowering as the presence of any floral structure (petals, sepals, stigma and stamen), and inflorescence for grasses, sedges and rushes, as used in Hart et al. (2023). Vascular plants were identified to species level by experts following Stace (2019), with the exception of *Taraxacum* microspecies and two aggregates ( *Festuca ovina* & *Festuca rubra* *= Festuca* spp.; *Phleum pratense* & *Phleum bertolonii* *= Phleum pratense s.l*). Experts also determined the broad habitat and NVC community visually in the field for each plot.

An expert manually checked the photos submitted by participants where the record did not match the expert field data. This was important for determining whether the species identification was correct but had been missed by the expert, or if the species was misidentified by the application leading to a false positive record i.e., incorrectly identifies the presence of a species that was actually absent from the plot.

### 2.3 Participant Feedback

To understand more about the participants' experience of using the application and potential future uses, we created a paper‐based questionnaire which was given to participants after completing their 25 m^2^ survey at the workshop (Approval from UKCEH Human Research Ethics Committee/09157). Informed consent was obtained from all participants. Participants' names were captured for the purpose of connecting their associated e‐Surveyor survey data; however, these were later coded and names were removed to anonymise the data.

The questionnaire was split into two sections (Appendix A). The first part was aimed at quantifying participants' prior knowledge and experience in recording plants and their familiarisation with the broad habitat classifications and the NVC. The second part was aimed at assessing the participants' experience of using the application. This included questions on how easy they found the survey to undertake and their confidence in the habitat output provided by the application, as well as free text boxes allowing the participant to expand on any issues, and if and where they might see themselves undertaking a survey using the application again in the future.

### 2.4 Data Analysis

To compare the total number of species recorded by participants and experts within the plots including false positives, we used a generalised linear mixed model (GLMM) with a Poisson distribution. Fixed effects comprised surveyor type (participant and expert) and habitat type (calcareous grassland, neutral grassland and improved grassland), whilst plot number was included as a random effect. We excluded five surveys which were carried out in the same plot by more than one participant (the first survey completed was selected). To understand if the participant's self‐assessed plant identification skills (derived from the questionnaire) influenced the number of species recorded, a separate GLMM was constructed with recording confidence as a fixed effect and plot number and habitat type as random effects. Plant identification skills were grouped as (1) those who had not participated in plant recording before or had low confidence or (2) medium or high confidence as determined from the questionnaire. All models were fitted using the ‘lme4’ package (Bates et al. 2014) in R version 4.4.0 (www.r‐project.org). Estimated marginal means were calculated using the ‘emmeans’ package (Lenth 2025), and model assumptions were checked using DHARMa diagnostics in the ‘DHARMa’ package (Hartig 2024).

To assess which plant species were accurately captured by participants during the workshops, we compared survey lists to the full inventory recorded by experts (expert data was compiled to generate one full list for each 25 m^2^ plot). Where species were consistent between participants and experts, these taxa were determined to be correct and individual photos captured through the application were not checked by experts. For species that were recorded by participants but did not match the expert data, an expert manually checked through photos submitted through the application to determine whether the species identification was correct but had been missed by the expert, or if the species identification was incorrect. For species identified incorrectly at the species level, we determined whether the species recorded had been identified correctly to the family level. Any remaining species were classified as ‘undetected’, that is, species that were recorded by the expert but were not captured either correctly or incorrectly by the participant. Photos where the target species was out of focus or unclear could not be compared with the expert data and were thus classified as incorrect (*n* = 27).

To understand factors influencing identification accuracy of plants known to be present, we ran a GLMM with a binomial distribution. Species which were identified correctly were allocated ‘1’, whilst those undetected by participants or recorded incorrectly on the application were allocated ‘0’. The fixed effects were included as an interaction: plant type (graminoid (grass/sedge/rush) or forb), whether the species was flowering (recorded by experts) and abundance (DAFOR score recorded by experts). Woody species (*n* = 10) were grouped into the forb category. Abundance data was amalgamated from the experts; where this was inconsistent for a given species within a plot, the highest abundance value was allocated. Few species were recorded as ‘dominant’ or ‘abundant’ (predominantly in the lower complexity habitats), thus these categories were combined with ‘frequent’ in the model. We excluded species correctly recorded only by participants, since there was no information on abundance or flowering status from experts. The participant ID and species were both included as random effects, since species may be recorded by multiple participants and thus have unique responses to the other interaction terms. We excluded plots that were surveyed twice by different participants (*n* = 5). The final model is as follows:

$$\begin{array}{c} {\text{Species}}^{'}\;\text{accuracy}\sim \text{Plant type}*\;\text{Flowering}*\;\text{Abundance}\\ \;+\left(1|\text{Participant}\;\mathrm{ID}\right)+\left(1|\text{Species}\right) \end{array}$$

To assess the accuracy of the determined broad habitat and NVC community provided by the application, we compared the top suggested habitat/NVC community and the top three suggested broad habitats and ten NVC communities with the habitat recorded by experts in the field. If the top two habitats had the same match score, and either one corresponded with the expert habitat, this was allocated as the correct top suggestion. The NVC was assessed at the community level (e.g., CG2), rather than the sub community level (e.g., CG2a, CG2b etc.), as recorded by the experts. Thus, for example CG2a and CG2b, would both be considered correct if the expert had classified the survey area as CG2. To understand whether the proportion of correctly identified species influenced the broad habitat classification accuracy on the application, we ran a Generalised Linear Model (GLM) with a binomial distribution. Surveys which correctly identified the first suggested habitat were allocated ‘1’, whilst those which matched with the second/third suggestion or not at all were allocated ‘0’. The proportion of expert‐identified species correctly identified in the 25 m^2^ plot was included as a fixed effect, which was centred and standardised by one standard deviation (Schielzeth 2010). We found that when we originally ran this model as a GLMM with participant ID as a random effect, very little of the variance was explained and led to overfitting (assessed by removing and adding the random effect), so we therefore excluded participant ID from the final model, a GLM. The ‘ggeffects’ package was used to generate predictions from the model (Lüdecke 2018).

## 3 Results

### 3.1 Number of Species

**FIGURE 2:**
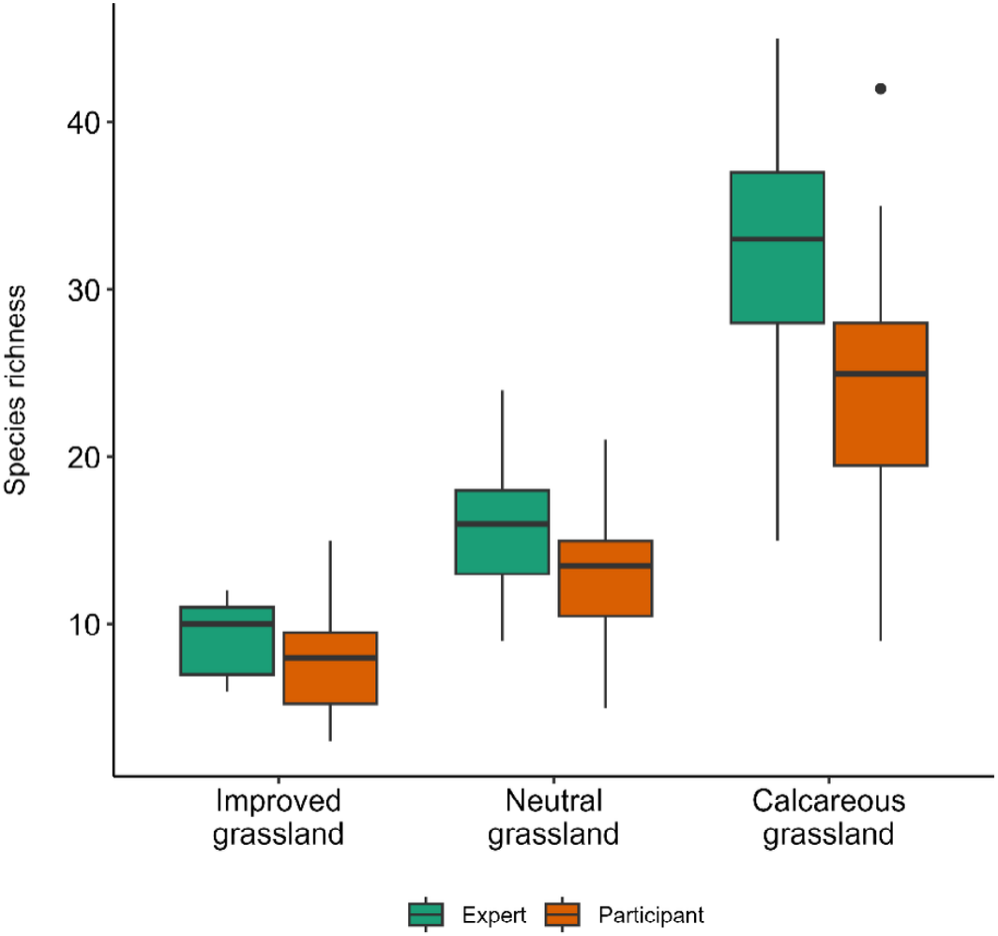
Boxplot of the number of plant species (including false positives) recorded per 25 m^2^ plot by participants (orange) and experts (green) for the three different habitat types during the e‐Surveyor workshops.

Thirty‐seven participants attended the e‐Surveyor workshops and completed a questionnaire, with 51 individual plant surveys submitted across the three habitats: calcareous grassland (*n* = 23), neutral grassland (*n* = 18) and improved grassland (*n* = 10). In total, 1178 individual species records were submitted on e‐Surveyor. Collectively, these records spanned across 246 species for participants, whilst experts recorded 139. At the plot level, participants recorded an average of 17.03 ± 1.21 (SE) species across all habitat types, whilst experts recorded 23.06 ± 1.06 per plot. The higher overall number of unique species captured by participants, despite fewer being recorded per plot, is because many of the records submitted by participants were false positives. Participants recorded the greatest number of species (including false positives) within calcareous grassland, followed by neutral grassland and improved grassland (Figures 2 and S1, Table S1). This pattern was also evident for the experts, reflecting the increased complexity of the grassland types, from improved grassland to calcareous grassland. This was supported by the model which showed there was strong evidence for a difference in the number of species recorded between the three habitat types (estimated marginal mean abs. diffs 8.4 ± 2.81 (95% CI), 14.1 ± 2.89, 27.9 ± 4.73 for improved, neutral and calcareous grassland respectively, *p* < 0.001). The participants identified fewer species per plot than the experts across all habitats (estimated marginal mean abs. diffs 16.6 v. 13.4, ratio 1.24 ± 1.15–1.35 (95% CI), *p* < 0.001). There was no evidence that the participants' plant recording skills influenced the number of species recorded on the application during a survey (−0.10 ± 0.10 (coefficient ± SE), 2.5% CI = −0.30, 97.5% CI = 0.10, *Z* = −0.98, *p* = 0.33; model AIC = 296.55, marginal *R* ^2^ = 0.01, conditional *R* ^2^ = 0.74; estimated marginal mean abs. diffs 14.4 v. 13.1, 1.1 ± 0.91–1.35, *p* = 0.325), which could be due to low power. DHARMa residual diagnostics can be found in Appendix B.

### 3.2 Species Identification Accuracy

**FIGURE 3:**
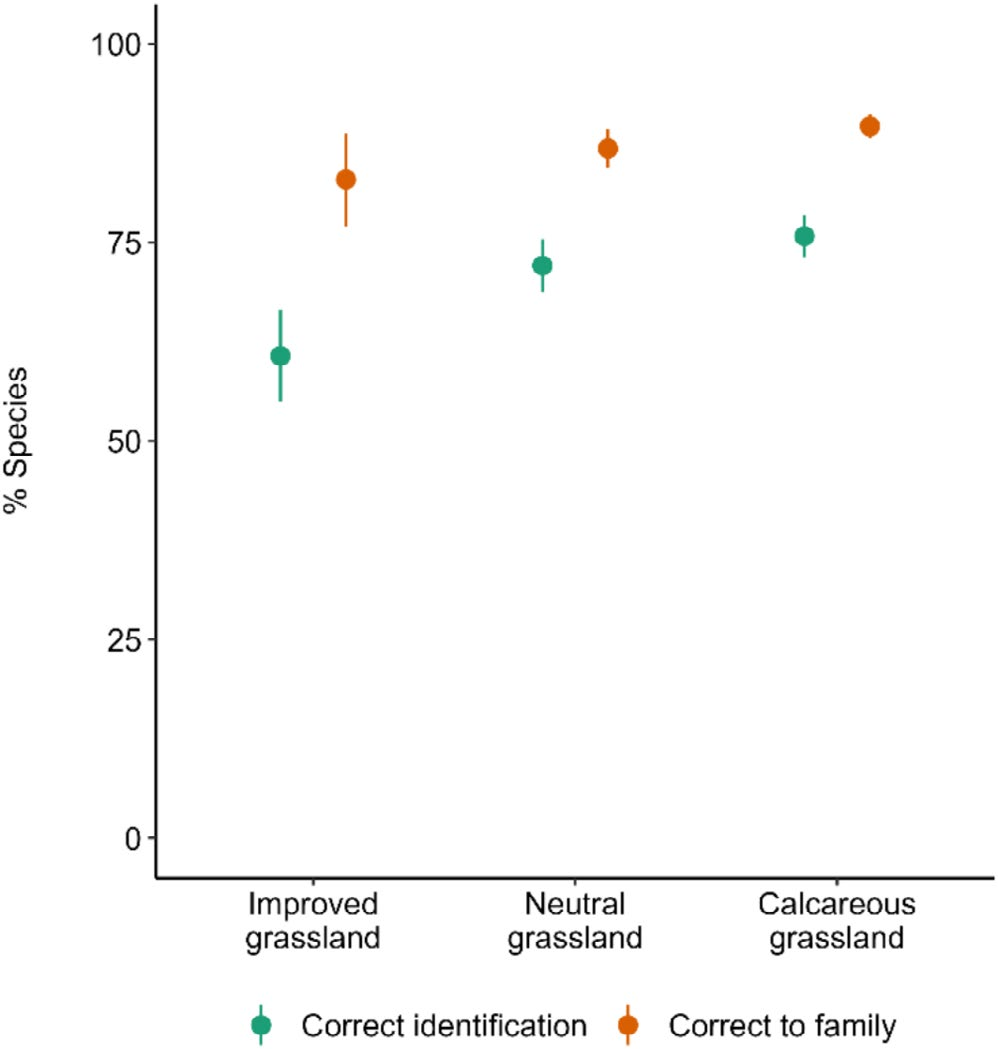
Mean (± standard error) percentage of species per 25 m^2^ plot that were identified correctly to species level, in addition to those that were also correct to family level within each of the three habitat types.

Of the species present that were recorded by participants, on average 71% were identified correctly to species level by the application across all surveys (Figure 3), where accuracy was assessed against the expert records. This accuracy increased to 87% when evaluating at the family level. The variation in accuracy across the three habitat types was small, with the highest accuracy evident in calcareous grassland.

**FIGURE 4:**
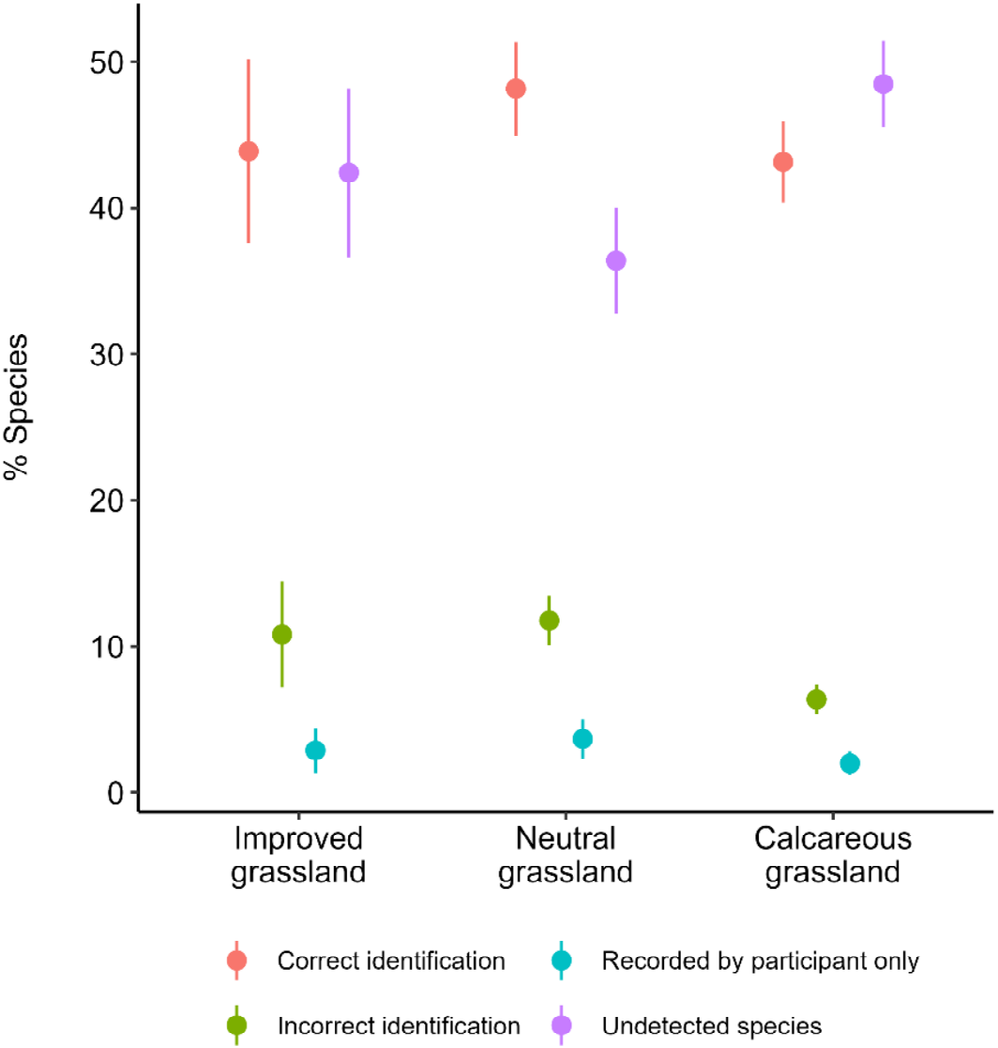
Mean (± standard error) percentage of species per 25 m^2^ plot that were correctly identified, incorrectly identified, undetected by participants and recorded only by the participants (not detected by expert) in each of the three habitat types.

On average, across all surveys, 45% of observable species were captured correctly to species level by participants, where observable species are those present in the plot according to the experts and also includes the small number detected by participants only. There was little variation in accuracy between the habitat types, with slightly higher accuracy (i.e., percentage of species correct) revealed for neutral grassland (48%) compared with improved (44%) and calcareous grassland (43%) (Figure 4). On average, 43% of observable species were undetected by participants during the survey, whilst 9% were identified incorrectly. A small number of species were recorded by participants and were not detected by any of the experts (3%). Fewer species were incorrectly identified for calcareous grassland, however more species went undetected during the survey in this habitat compared with neutral and improved grassland.

**FIGURE 5:**
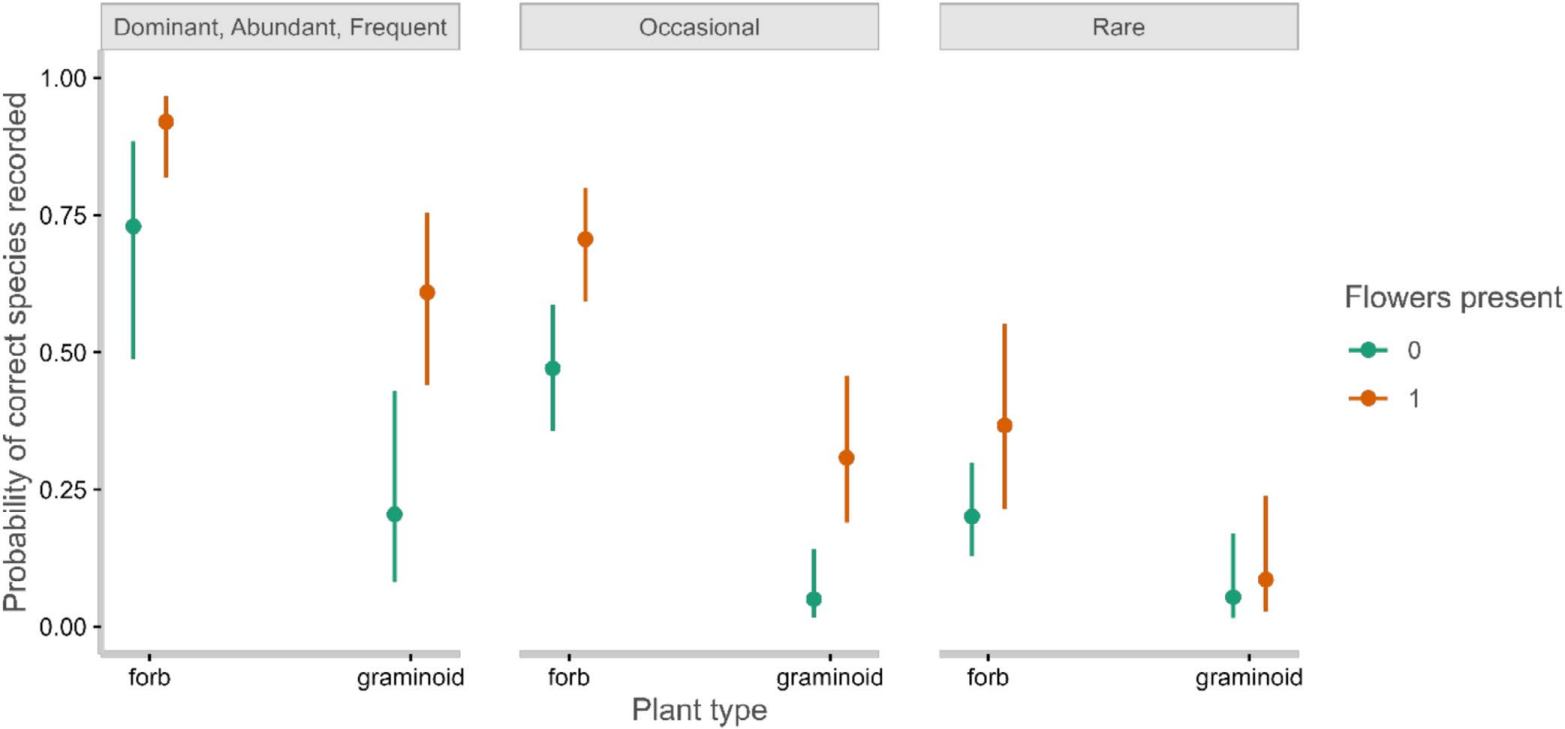
The effect of plant type (forb, graminoid), flowering (1 = flowers present, 0 = flowers absent) and abundance (‘Dominant, Abundant, Frequent’, ‘Occasional’ and ‘Rare’) on the likelihood of a plant species being captured correctly by a participant using the e‐Surveyor application.

The GLMM suggested that the type of plant recorded (graminoid or forb; *p* = 0.002), whether the species was flowering (*p* = 0.029) and its abundance (*p* = 0.002, DAF vs. R) were important factors for capturing a species correctly using the e‐Surveyor application (Table S2). Participants were more likely to record a species correctly where the plant was more abundant, flowering and a forb or woody species rather than a grass, sedge or rush (Figure 5), however, there was no evidence for an interaction between these factors, which may be due to the low power (Table S2). DHARMa residual diagnostics can be found in Appendix C.

### 3.3 Habitat Accuracy

**FIGURE 6:**
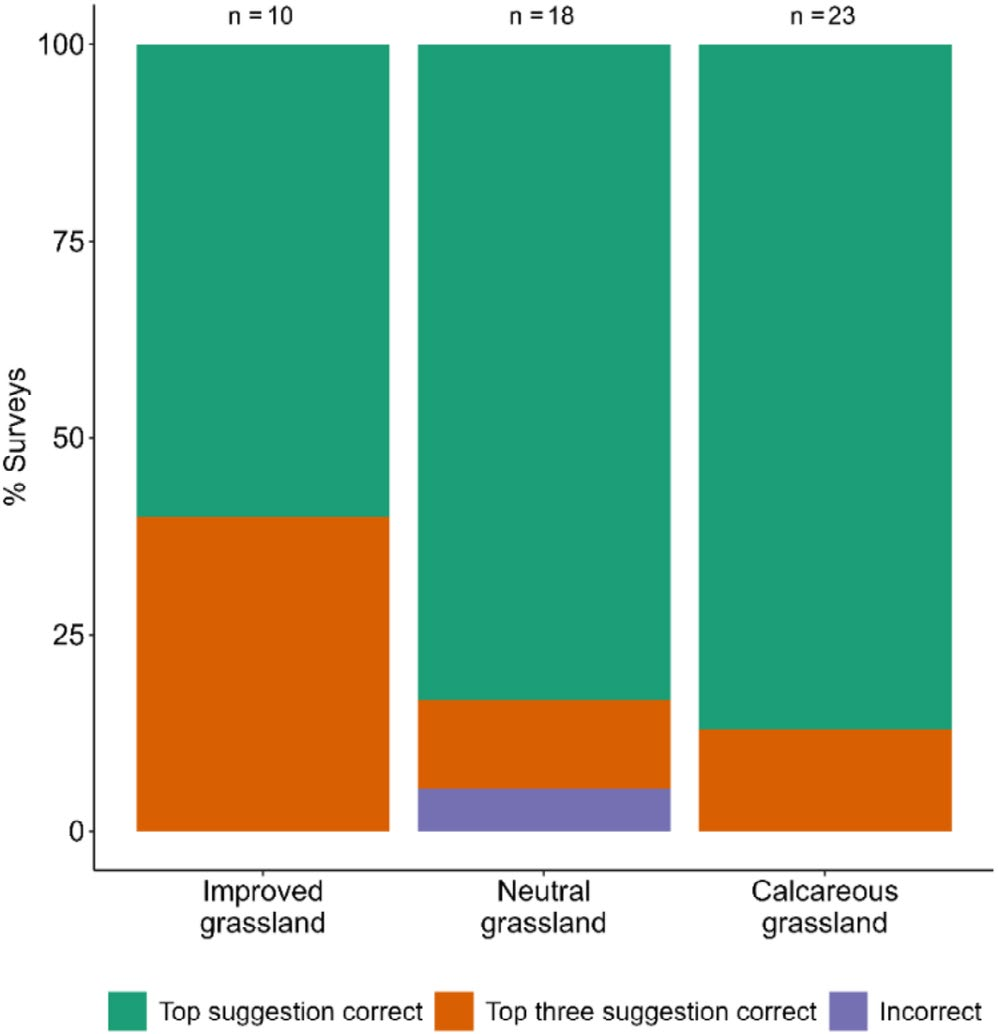
Percentage of surveys where the first broad habitat suggestion and top three broad habitat suggestions on the application correctly identified the habitat determined by the experts in the field for each of the three habitat types.

**FIGURE 7:**
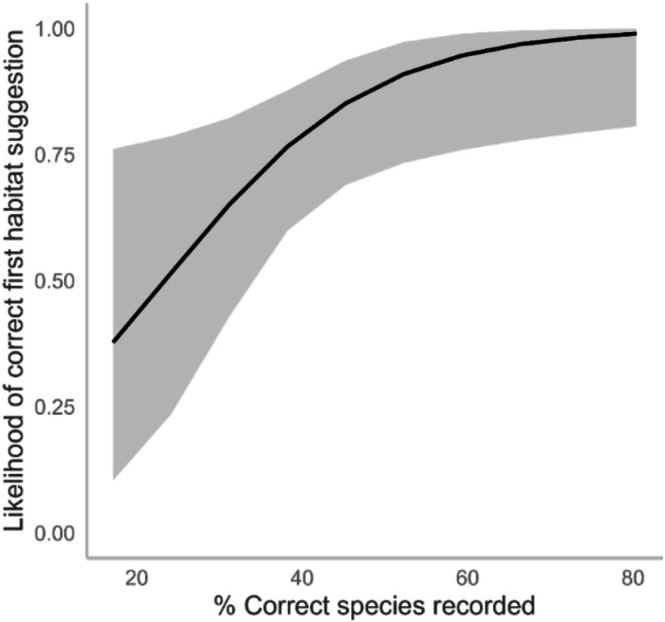
The relationship between the percentage of species recorded accurately and the likelihood of a participant obtaining the correct first suggested broad habitat on the e‐Surveyor application. Shaded areas show confidence intervals for the fixed effect.

Eighty percent of surveys identified the correct broad habitat according to the experts when evaluating the top broad habitat suggestion on the application (Figure 6a). This increased to 98% when considering the top three habitat suggestions. Calcareous grassland was the most correctly identified first broad habitat suggestion. Only one survey (neutral grassland) did not match with any of the top three suggested broad habitats. There was good evidence to suggest that the likelihood of obtaining the correct first broad habitat suggestion increased with the proportion of correctly identified observable species (1.12 ± 0.51 (coefficient ± SE), 2.5% CI = 0.12, 97.5% CI = 2.12, *Z* = 2.20, *p* = 0.028; model AIC = 43.13, Cragg‐Uhler *R* ^2^ = 0.21, McFadden *R* ^2^ = 0.14; Figure 7).

A quarter of the surveys identified the correct first NVC community suggested by the application according to the NVC community designated by the expert in the field. This increased to 71% when considering the ten NVC community suggestions (including the first) on the application. The improved grassland community (MG7) was the most accurately identified first suggestion, whilst improved grassland and calcareous grassland communities performed more accurately across all ten suggestions on the application (Table 1). DHARMa residual diagnostics can be found in Appendix D.

**TABLE 1:** Percentage of surveys where the top NVC community suggestion and top ten NVC community suggestions on the application correctly identified the NVC community determined by the experts in the field summarised for each of the three habitat types. The number of surveys undertaken in each NVC community is indicated in brackets.

Habitat type	NVC community (no. of surveys)	Top suggestion	Top 10 suggestions
Calcareous grassland	CG2 *Festuca ovina—Avenula pratensis* grassland (6) CG3 *Bromus erectus* grassland (17)	13%	91%
Neutral grassland	MG1 *Arrhenatherum elatius* grassland (4) MG4 *Alopecurus pratensis* — *Sanguisorba officinalis* grassland (2) MG5 *Cynosurus cristatus* — *Centaurea nigra* grassland (6) MG6 *Lolium perenne* — *Cynosurus cristatus* grassland (6)	22%	40%
Improved grassland	MG7 *Lolium perenne* leys and related grasslands (10)	60%	90%

### 3.4 Participant Feedback

**FIGURE 8:**
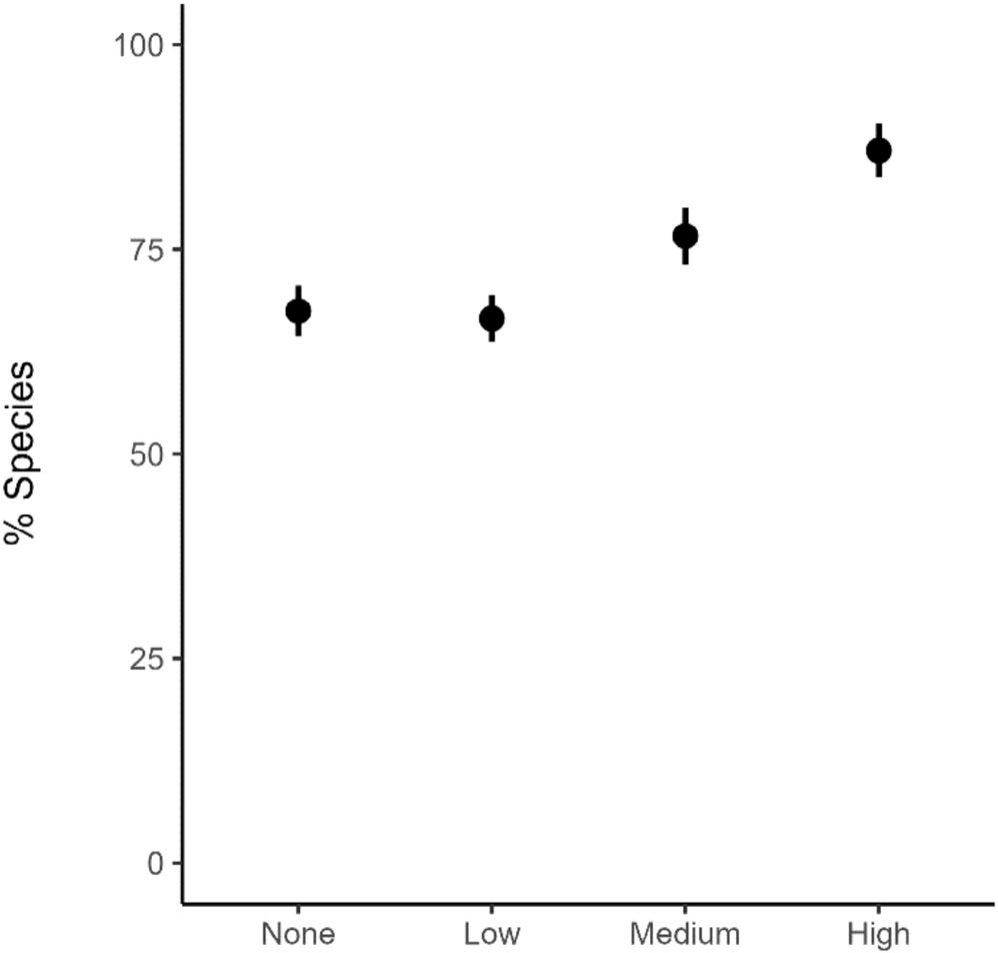
Mean (± standard error) percentage of species per 25 m^2^ plot that were correctly identified across the participants' confidence levels for plant recording (none (*n* = 8), low (*n* = 20), medium (*n* = 15) and high (*n* = 3)).

**FIGURE 9:**
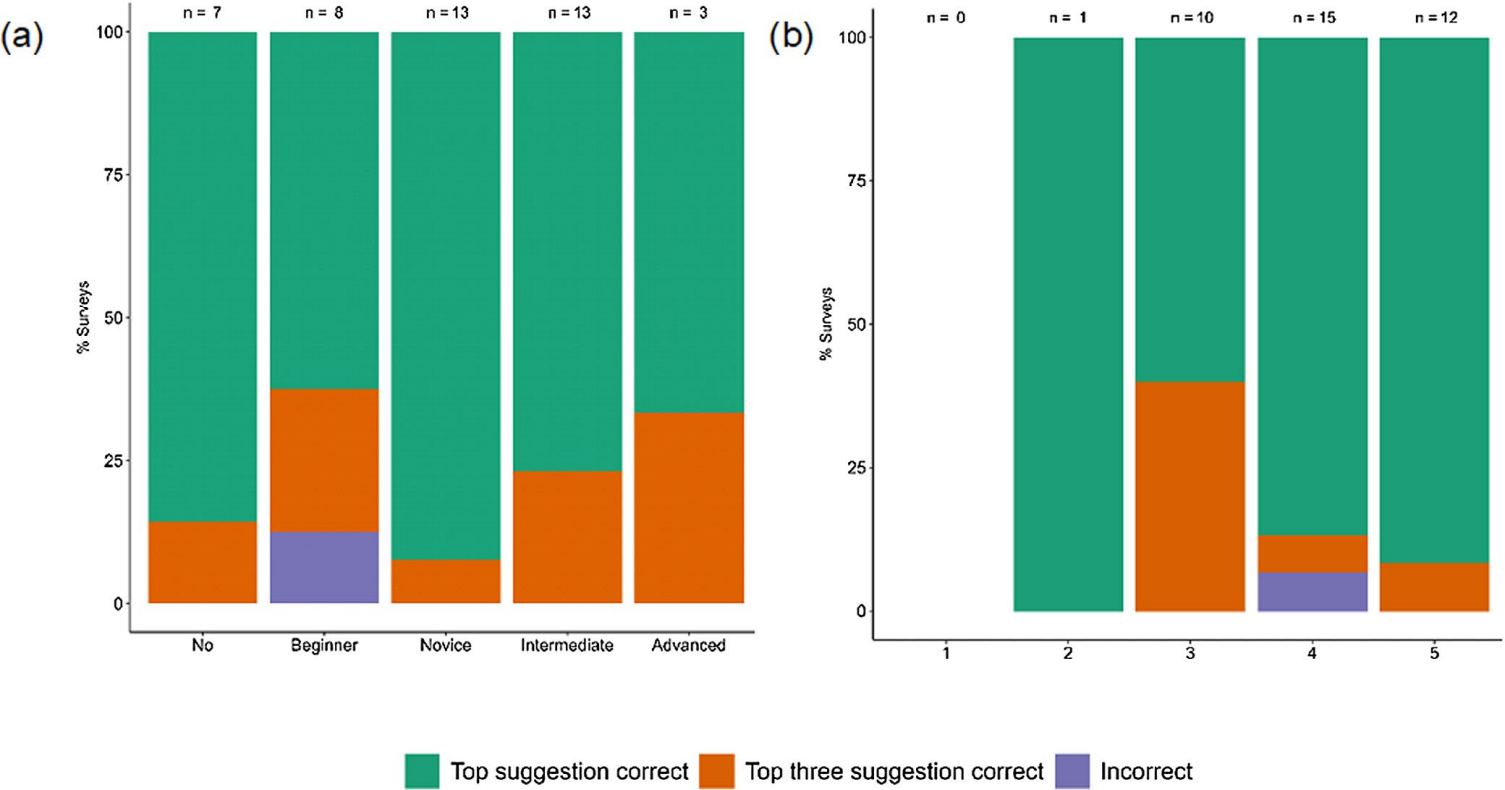
Percentage of surveys where the first broad habitat suggestion and top three broad habitat suggestions on the e‐Surveyor application correctly identified the habitat determined by the expert across (a) the familiarity of the participant with the broad habitat classification system and (b) their confidence in the broad habitat output provided on the e‐Surveyor application, where 1 is low confidence and 5 is high.

Most participants had low (*n* = 14) or medium (*n* = 12) confidence in botanical recording, three participants stated high confidence, whilst eight participants didn't currently participate in recording plants. Of those species that participants recorded during the survey, good species identification accuracy was evident across all plant recording abilities, albeit slightly lower for those who do not currently participate in any recording (Figure 8). Most participants (68%) did not manually enter species; however, a few participants corrected the AI suggestion if they believed it to be wrong, though this occurred regardless of the participant's plant recording ability. More participants were familiar with the broad habitat classification system compared with the NVC (Table 2). The percentage of surveys that correctly identified the first and top three habitat suggestions was largely similar across all levels of participants' broad habitat familiarity, with the only incorrect habitat type arising for a beginner (Figure 9a). Participants that were more confident in the accuracy of the broad habitat output presented on the e‐Surveyor application tended to obtain the correct first habitat suggestion (Figure 9b).

**TABLE 2:** Number of participants (total *n* = 37) and their reported knowledge and familiarity with broad habitats and the National Vegetation Classification. See Appendix A for interpreting familiarity levels.

Familiarity level	Broad habitat	NVC
Advanced	3	2
Intermediate	9	4
Novice	10	8
Beginner	8	6
Not familiar	7	17

Most participants had not used the e‐Surveyor application prior to attending the workshop (78%). Despite this, most participants found the application easy to use (81% scored 4 or 5, where 5 = very easy), though most experienced some kind of difficulty during the workshop (70%). This was largely due to difficulties obtaining internet access which is required to receive the species suggestions and view the final habitat report on the application. However, the photos are stored on the system, and species suggestions can be retrieved as soon as there is an internet connection again. Other minor issues specific to an individual's phone occurred, including differences between Android and iOS, camera issues and a faulty GPS ‘location not found’. Issues were also raised on how instructions on the application could be improved, for example, ‘buttons not sensitive’ and ‘didn't know how to delete species’. Regardless of the issues, all participants enjoyed using the application (scoring 4 or 5 out of 5), 97% would use it again and all would recommend to others. Participants described using the application in the future for personal use, and professionally for managing and monitoring local conservation sites ‘local meadow I manage’ and ‘on my grassland … and through work helping farmers’. Participants also stated that the application would help to improve their knowledge of habitats, whilst one participant noted using it for reassurance when undertaking their own independent surveys in the future.

## 4 Discussion

By undertaking workshops in the field with citizen scientists, we demonstrate that the e‐Surveyor mobile application is a useful tool for supporting citizen scientists to undertake biological recording for plant identification and determining habitat type. As expected, participants in our workshops recorded fewer plant species compared to experts, which is consistent with other citizen science studies in the literature (Fitzpatrick et al. 2009, Kallimanis et al. 2017). On average nearly half of the observable species were accurately captured by participants, whilst the remaining species were often undetected rather than recorded incorrectly. A small number of truly present species were only recorded by participants. Whilst some of these were genuinely missed by experts, highlighting the challenge of surveying in complex habitats like calcareous grassland, other examples were the result of a mismatch, where a participant had recorded outside of the designated 25 m^2^ plot. Of the species that were captured on the application by participants, on average 71% were identified correctly to species level. Given that very few recorded species were entered manually during the workshops, this suggests the automated identification performed well and is comparable to other studies, even though these were not carried out in the field (August et al. 2020; Hart et al. 2023; Jones 2020). Even higher accuracy was revealed with decreasing taxonomic resolution (family level = 87%), as found in August et al. (2020).

This study showed that species were more likely to be accurately identified where the plant was more abundant, flowering and a forb (herbaceous plant) rather than a graminoid, which is consistent with other plant detectability studies (Garrard et al. 2013; Hauser et al. 2022; McCarthy et al. 2013; Perret et al. 2023). Plants that are more abundant are easier to detect and offer more opportunity to obtain the most suitable image for automated identification. Similarly, those in flower are more likely to be noticed in a complex sward. This is consistent with Marcenò et al. (2021), who compared botanical data collected by Facebook group members with data collected by scientists in 6366 vegetation plots across Sicily. Plants recorded within the Facebook group tended to be showier and more colourful compared with those collected by scientists. Forbs were more likely to be identified correctly in this study, as seen in Hart et al. (2023). They assessed the accuracy of five plant identification applications and revealed that these were less confident for rushes, sedges and grasses relative to forbs and woody species. This is likely because forbs are easier to detect and photograph than grasses, sedges or rushes, which have a very fine structure and are visually similar. Furthermore, the AI technology is more often trained using a greater number of images for forbs compared with graminoids. Some species groups within forbs and graminoids may be more difficult to identify through the app than others; for example, some Asteraceae can appear very similar to one another, and finer grasses can be more difficult to photograph than coarser grasses. It would be important to test which species groups are more consistently identified with lower accuracy to refine AI model development; however, we were unable to test this statistically here due to small sample sizes.

Eighty per cent of surveys identified the correct broad habitat during the workshops. This rose to 98% when the top three suggested habitats on the application were considered. The correct top broad habitat was more likely to be achieved when a greater proportion of correctly identified plants were captured during a survey. Despite this, our results also show that participants were often still able to obtain accurate broad habitat associations on the application, even when less than half of observable species in a plot were captured or the number of false positive species were high. This is because many of the false positive species recorded by participants were non‐native species (e.g., *Bothriochloa ischaemum, Lepidium hirtum, Acaena ovalifolia*) that didn't have an associated broad habitat type in PlantAtt, and thus would not have contributed towards the habitat match score. The ability to obtain accurate habitat associations without a full species list is supported by evaluation of the NPMS, where indicator species were able to capture information on ecological and environmental gradients (Pescott et al. 2019). Calcareous grassland was determined correctly more often than improved and neutral grassland, most likely because this habitat contains many unique specialist species that are not associated with other grassland types. Despite this, there was some confusion between calcareous grassland and neutral grassland, since some survey locations were in poorer condition, thus containing less typical specialist species and instead more species associated with both habitat types (e.g., *Plantago lanceolata* , *Lotus corniculatus* ). Degraded calcareous grassland can support assemblages containing a higher proportional of neutral grassland indicators, even though the substrate is still calcareous. Thus, trying to assign a broad habitat type (physiognomic habitat classification) using species compositional data can lead to poorer fits, especially for degraded or marginal habitats, ecotones, and transitional communities (Song et al. 2024). The selection of an appropriate survey area has important implications for users outside of this current study where the plot is not predefined. Since this study, we have enhanced our user guidance to direct a user to select their survey area within homogeneous vegetation. This is important for obtaining a more reliable habitat report, where vegetation communities found within different habitats are not confused.

The derived NVC community was less accurate compared with the broad habitats, which is to be expected given the complexity of this classification system. Furthermore, a full list of observable species is critical for determining NVC community unlike the broad habitat associations (Rodwell 2006), yet this was not achieved across any of the surveys in this study. The accuracy increased when the top ten NVC communities were considered, however there were too few surveys undertaken in some NVC communities for a reliable assessment. Calcareous grassland NVC communities were typically identified more accurately when considering the top ten NVC communities, particularly when the presence of a key determinant species was successfully recorded by a participant (e.g., the grass *Bromopsis erecta* for CG3 *Bromus erectus* grassland). The suggested top ten NVC communities should not be used in isolation to determine the final NVC community, but instead alongside additional resources such as field guides and keys. The NVC functionality in e‐Surveyor is likely to be too complex for most citizen scientists, however it may be useful for nature reserve managers undertaking assessments of condition on statutory sites (JNCC 2004) or other ecologists aspiring to learn more about the NVC.

All participants enjoyed using the e‐Surveyor application during the workshops and found it easy to use. This is important to understand, since these factors are more likely to encourage a citizen scientist to use the application again. It also helps to inform future changes in the application and provides a useful template for other applications/tools. Participants reported using e‐Surveyor for personal interest in the future, but also for recording more formally under a professional or volunteer capacity on local nature reserves and wildlife sites, as well as using the application to improve their knowledge of broad habitats more generally. Information on how specific functions could be improved, where extra interpretation and new features could be added was also obtained from the questionnaire. This information is critical for ensuring automated identification applications, such as e‐Surveyor, are designed to be as informative and functional as possible. This feedback is addressed in the latest release of the e‐Surveyor application.

Despite an individual's confidence in plant recording and familiarisation with broad habitats, the majority were able to accurately identify a good number of species and determine the correct broad habitat. This suggests that the e‐Surveyor application is a useful tool for supporting biological recording. This could include the generation of plant species lists for important nature sites or the assessment of habitat condition through understanding key indicator species present. For those with more experience, the application may be useful for supporting identification and narrowing down where to look in comprehensive field guides and taxonomic keys. Furthermore, the habitat suggestions may provide a second opinion or offer reassurance to the user. On the other hand, for inexperienced citizen scientists, e‐Surveyor provided an opportunity to gain knowledge and confidence in plant recording, which may lead to greater engagement with nature more broadly (Day et al. 2022). The e‐Surveyor application has several advantages over using typical plant identification applications such as Flora Incognita. Firstly, it allows structured botanical recording, where records are stored on a web application, allowing users to view their data on a map and export species lists as required. This enables users to repeat their survey monitoring, which is important for assessing trends in biodiversity and the effectiveness of conservation management. Secondly, e‐Surveyor provides useful interpretation of the plant survey, including associated habitats as demonstrated in this study, but also information on associated pollinators and crops that may be supported by these. It is important to remember that automated identification applications, such as e‐Surveyor, are not intended to replace traditional biological recording by experts; however, this study shows they can be a useful tool for supporting such activities, as well as improving confidence, knowledge, and engagement. By testing such applications with citizen scientists, we have a greater understanding of their capability, limitations, and potential future uses.

## Author Contributions

**Lucy E. Ridding:** conceptualization (equal), data curation (equal), formal analysis (lead), investigation (lead), methodology (lead), project administration (lead), visualization (lead), writing – original draft (lead), writing – review and editing (lead). **Morag McCracken:** formal analysis (supporting), methodology (supporting), writing – review and editing (supporting). **Zephyr Orsler:** formal analysis (supporting), visualization (equal), writing – review and editing (equal). **Emily V. Upcott:** investigation (equal), writing – review and editing (equal). **Nadine Mitschunas:** investigation (equal), writing – review and editing (supporting). **Karolis Kazlauskis:** software (equal), writing – review and editing (supporting). **Zeke Marshall:** software (equal), writing – review and editing (supporting). **Grace Skinner:** software (equal), writing – review and editing (supporting). **Simon M. Smart:** methodology (supporting), writing – review and editing (supporting). **Colin A. Harrower:** methodology (supporting), writing – review and editing (supporting). **Oliver L. Pescott:** methodology (supporting), writing – review and editing (supporting). **Richard F. Pywell:** conceptualization (equal), writing – review and editing (supporting). **Tom A. August:** conceptualization (equal), methodology (equal), software (supporting), writing – review and editing (equal).

## Funding

This work was supported by the Department for Environment, Food and Rural Affairs, UK Government, Natural Capital and Ecosystem Assessment programme; Centre for Ecology and Hydrology (Grant NE/W005050/1).

## Conflicts of Interest

The authors declare no conflicts of interest.

## Supporting information


**Data S1:** Supporting Information.

**Figure S1:** Scatterplot showing the number of plant species recorded per 25 m^2^ plot by participants and experts for the three different habitat types during the e‐Surveyor workshops. Only a single participant's data is used per plot.
**Table S1:** Parameter estimates and confidence intervals of generalised linear mixed models (poisson) comparing the numbers of species identified by user types (expert and participant) in broad habitat types (improved, neutral and calcareous grasslands) as additive terms. Model AIC = 893.20, marginal *R* ^2^ = 0.75, conditional *R* ^2^ = 0.82. Random effects plot std. dev. = 0.16. 46 plot groups.
**Table S2:** Parameter estimates and confidence intervals of generalised linear mixed models (binomial) of the likelihood of species being correctly recorded on the e‐Surveyor application with plant type, flowering and abundance as interaction terms. Model AIC = 1334.44, marginal *R* ^2^ = 0.26, conditional *R* ^2^ = 0.50. Random effects species std. dev. = 1.10, app user std. dev. = 0.65. 139 species groups, 46 plot groups.
**Appendix A:** Questionnaire completed by participants after completing an e‐Surveyor habitat survey at one of the workshops.
**Appendix B:** DHARMa residual diagnostics: number of species.
**Appendix C:** DHARMa residual diagnostics: species identification accuracy.
**Appendix D:** DHARMa residual diagnostics: habitat accuracy.

## Data Availability

The data used in this study is published and freely available via the Environmental Information Data Centre (EIDC): Ridding et al. (2025).
