# Supplementary material for: Evaluation of the e‐Surveyor Mobile Application for Undertaking Plant Surveys and Predicting Habitat Type
Source: Ecol Evol. 2026 Jun 29;16(7):e73901. doi: 10.1002/ece3.73901 (PMC13314545; doi:10.1002/ece3.73901)

Plant surveys and questionnaire evaluation of the e-Surveyor mobile application

Lucy E Ridding, Morag McCracken, Zephyr Orsler, Emily V Upcott, Nadine Mitschunas, Karolis Kazlauskis, Zeke Marshall, Grace Skinner, Simon M Smart, Colin A Harrower, Oliver L Pescott, Richard F Pywell and Tom A August

# Summary

Vascular plant and questionnaire data from 51 surveys, used to assess the functionality of the e-Surveyor mobile application. Vascular plants were recorded by 37 volunteer participants at workshops using the habitat survey within e-Surveyor. Participants were able to utilise the inbuilt AI technology to generate species suggestions, otherwise they had the option to record a plant species manually if they were confident with the identification. Plants were recorded within a 25 m^2^ plot in calcareous, neutral or improved grassland. Each plot recorded by a participant was also surveyed by 2-3 professional expert botanists that worked independently to collect a full plant inventory list. Participants completed a paper-based questionnaire after their survey which was used to assess the participants’ experience of using the application. Workshops were carried out between June and August 2024 in Central and Southern England.

# File format and names

The following files are provided in comma-separated values (CSV) format:

| Name | Description |
| --- | --- |
| Survey_metadata.csv | Provides information on the volunteer and plot IDs, as well as the date of the survey. |
| Questionnaire_data.csv | Participant responses to the questionnaire |
| Volunteer_data.csv | Plant survey data collected by the participants |
| Expert_data.csv | Plant survey data collected by the experts |

# Nature and units of recorded values

Please note all CSV files can be joined using the “unique_plot_id” column ID.

Survey metadata.csv:

| **Column_ID** | **Description** |
| --- | --- |
| volunteer_ID | Unique ID given to each participant |
| survey_ID | Indicates where a participant recorded more than one survey |
| unique_plot_ID | A unique identifier for a survey undertaken by a participant |
| duplicate | Indicates where two participants recorded within the same plot |
| broad habitat type | Habitat type survey was carried out in: improved grassland, neutral grassland and calcareous grassland |
| date | Date of survey |

Questionnaire data.csv:

| **Column ID** | **Question and answer format** |
| --- | --- |
| unique_plot_ID | A unique identifier for a survey undertaken by a participant |
| 1 | Q: How confident are you in your identification skills for plants? A:  Low confidence – I consider myself to be a beginner recorder but do have some experience;  Medium confidence – I consider myself to be an intermediate level recorder;  High confidence - I could be described as an expert recorder;  I don’t currently participate in recording plants |
| 2 | Q: How familiar are you with Broad Habitat classifications? A:  Not familiar at all;  Beginner (I have very little knowledge);  Novice (I have some knowledge and have used a few times);  Intermediate (I have some knowledge and have practical experience);  Advanced (I have good knowledge and have used a lot);  Expert (I have expert knowledge and use widely); |
| 3 | Q: How familiar are you with the National Vegetation Classification (NVC)? A:  Not familiar at all;  Beginner (I have very little knowledge);  Novice (I have some knowledge and have used a few times);  Intermediate (I have some knowledge and have practical experience);  Advanced (I have good knowledge and have used a lot);  Expert (I have expert knowledge and use widely); |
| 4 | Q: Have you used the e-Surveyor app before today? A: Yes or No |
| 4a | Q: If yes, which survey. A: Identify plant; Record a habitat; Structured survey; Farmland Carabids; Moth recording |
| 5 | Q: On a scale of 1-5, how easy did you find undertaking the “record a habitat” survey on the app (1 = very difficult, 5 = very easy). A: 1, 2, 3, 4 or 5 |
| 6a | Q: Which of the following plants did you tend to take photos of a) Plants that were flowering which you recognised. A: y |
| 6b | Q: Which of the following plants did you tend to take photos of b) Plants that were flowering which you did not recognise. A: y |
| 6c | Q: Which of the following plants did you tend to take photos of c) Plants that were not flowering that you recognised. A: y |
| 6d | Q: Which of the following plants did you tend to take photos of d) Plants that were not flowering that you did not recognise. A: y |
| 6e | Q: Which of the following plants did you tend to take photos of e) Everything. A: y |
| 6f | Q: Which of the following plants did you tend to take photos of f) Other (Please state. A: Free text |
| 7a | Q: Did the app struggle to identify any of your plant photos? A: Yes or No |
| 7b | Q: If so, how many? A: Free text |
| 8a | Q: Did you enter any species in your survey manually (i.e. without taking a photo and instead holding down the plus button)? A: Yes or No |
| 8b | Q: Please state which. A: Free text |
| 9 | Q: Following completion of the survey how confident do you feel that the app provided the correct Broad Habitat type? (1 = not at all confident, 5 = very confident). A: 1, 2, 3, 4, 5, N/A |
| 10 | Q: Following completion of the survey how confident do you feel that the app provided the correct National Vegetation Classification? (1 = not at all confident, 5 = very confident). A: 1, 2, 3, 4, 5, N/A |
| 11 | Q: How long did the survey take you to complete on the app? A: < 5 minutes; 5 – 10 minutes; 10 – 20 minutes; 20 – 30 minutes; >30 minutes |
| 12 | Q: Did you experience any difficulties using the app? (e.g. any error messages or functions that didn’t work etc.). A: Yes or No |
| 13 | Q: If yes, please let us know what these were. A: Free text |
| 14 | Q: On a scale of 1-5, how enjoyable did you find using the app (1 = not very enjoyable, 5 = very enjoyable). A: 1, 2, 3, 4 or 5 |
| 15 | Q: Would you use the app in the future? A: Yes or No |
| 16 | Q: If you answered yes, please let us know where and how you could see yourself using the app A: Free text |
| 17 | Q: If you answered no, please could you elaborate. A: Free text |
| 18 | Q Would you recommend the app to others? A: Yes or No |
| 19 | Q: Please let us know in free text below if you have any further comments. A: Free text |

Volunteer data.csv:

| **Column_ID** | **Description** |
| --- | --- |
| unique_plot_ID | A unique identifier for a survey undertaken by a participant |
| species_name | Species name provided from e-Surveyor output |
| common_name | Common name provided from e-Surveyor output |

Please note a species may be recorded more than once within a survey, if a participant photographed different individual plants, that were in fact the same species.

Expert data.csv:

| **Column_ID** | **Description** |
| --- | --- |
| unique_plot_ID | A unique identifier for a survey undertaken by a participant |
| recorder/flowers | Identifies which recorder surveyed the plot and whether the species was in flower. Note the flowering status was contributed by all experts. |
| species name (*Achillea millefolium* to *Viola riviniana*) | Provides the DAFOR (Dominant, Abundant, Frequent, Occasional or Rare) score for each vascular plant species. For “Flowers”, 1 indicates the species was flowering in the survey plot. |

# Spatial coverage Central and Southern England

# Temporal coverage and resolution June – August 2024

# Methods

*Background to e-Surveyor mobile application*

e-Surveyor is a free mobile application developed by the UK Centre for Ecology & Hydrology; it allows farmers, landowners, and citizen scientists to assess the quality of the habitats they manage and have an interest in conserving (<https://esurveyor.ceh.ac.uk/>). This dataset used the habitat survey function, which allows citizen scientists to generate a plant species list by manually entering known plant species or by using the in-built AI technology to generate species suggestions, which are provided by the Pl@ntNet API (Affouard et al., 2017); the API uses Kew Royal Botanical Gardens’ Plants Of The World Online (POWO) checklist as the taxonomic backbone. For each species suggestion Pl@ntNet provides a confidence score (percentage). One photo is required, however up to five photos can be added per individual plant, which may increase the confidence score. The species with the highest confidence score is automatically selected by the application, however users can override this selection if they disagree with the suggestion.

*Field workshops*

To test the habitat functionality in the e-Surveyor application we ran seven field workshops with different citizen science groups ranging from 4-10 participants in size. Participants were those with an interest in biological recording, wildlife applications or were members of a practical conservation group. Workshops took place in central and southern England between June and August 2024 to coincide with the peak flowering period in England, since flowers are a key identifying feature and are generally easy to photograph using a camera phone. Workshops were held in three different habitat types ranging from high to low complexity based on the expected number of species present: calcareous grassland (UKHab = g2), neutral grassland (g3) and improved grassland (g4).

At each workshop, participants were introduced to e-Surveyor before being asked to undertake a “Record a habitat” survey on the application. We instructed participants to record all vascular plants within a 25 m^2^ plot. This plot size was selected to ensure participants had sufficient vegetation of interest to explore, particularly in the lower complexity habitats. Furthermore, this plot size is also consistent with plot sizes used in other citizen science recording schemes e.g. the National Plant Monitoring Scheme (NPMS) (Pescott et al., 2019). Participants were able to utilise the camera function with the inbuilt AI technology to generate species suggestions, otherwise they had the option to record a plant species manually if they were confident with the identification. Participants were able to take up to five photos per individual plant and could override species suggestions thought to be incorrect. Each participant recorded their own 25 m^2^ plot independently, though a small number of plots were recorded by two participants.

Each plot recorded by a participant was also surveyed by 2-3 professional expert botanists that worked independently to ensure an accurate full inventory list was collated. Only a small number of plots were surveyed by one expert, predominantly in the simplest habitat (improved grassland). Experts recorded a simple abundance using the DAFOR scale (Dominant, Abundant, Frequent, Occasional or Rare) for each vascular plant present, and recorded whether it was flowering. We defined flowering as the presence of any floral structure (petals, sepals, stigma and stamen), and inflorescence for grasses, sedges and rushes, as used in Hart et al. (2023). Vascular plants were identified to species level by experts following Stace (2019), with the exception of *Taraxacum* microspecies and two aggregates (*Festuca ovina* & *Festuca rubra = Festuca* spp.; *Phleum pratense* & *Phleum bertolonii = Phleum pratense s.l*).

*Participant feedback*

To understand more about the participants’ experience of using the application and potential future uses, we created a paper-based questionnaire which was given to participants after completing their 25 m^2^ survey at the workshop (Approval from UKCEH Human Research Ethics Committee/09157). Informed consent was obtained from all participants. Participants’ names were captured for the purpose of connecting their associated e-Surveyor survey data, however these were later coded and names were removed to anonymise the data.

The questionnaire was split into two sections (see Appendix A)). The first part was aimed at quantifying participants’ prior knowledge and experience in recording plants and their familiarisation with the broad habitat classifications (JNCC, 2019) and the National Vegetation Classification (Rodwell, 1998). The second part was aimed at assessing the participants’ experience of using the application. This included questions on how easy they found the survey to undertake and their confidence in the habitat output provided by the application, as well as free text boxes allowing the participant to expand on any issues, and if and where they might see themselves undertaking a survey using the application again in the future.

# Quality control

Expert data were collected by the same 2-3 experienced field ecologists. Paper data sheets were digitised into Excel and were checked for any anomalies.

# Miscellaneous

For the full details of this study please see:

Ridding et al., (2025) Evaluation of the e-Surveyor mobile application for undertaking plant surveys and predicting habitat type

# Funding

This work was funded through a research partnership agreement between the Natural Capital and Ecosystem Assessment programme (NCEA Defra) and the UK Centre for Ecology & Hydrology (UKCEH) number C21333, which builds upon work supported by the Natural Environment Research Council (NERC) award number NE/W005050/1 as part of the research programme AgZero+: Towards sustainable, climate-neutral farming. TAA was also supported by funding from the Natural Environment Research Council (NERC) under research programme ACCESS-UK NE/Y006208/1.

# References

Affouard, A., Goeau, H., Bonnet, P., Lombardo, J.-C., & Joly, A. (2017). PL@NTNET APP IN THE ERA OF DEEP LEARNING. In ICLR: International Conference on Learning Representations. [www.tela-botanica.org/appli:identiplante](http://www.tela-botanica.org/appli:identiplante)

JNCC. (2019). UK BAP Priority Habitats. <Https://Jncc.Gov.Uk/Our-Work/Uk-Bap-Priority-Habitats/>.

Pescott, O. L., Walker, K. J., Harris, F., New, H., Cheffings, C. M., Newton, N., Jitlal, M., Redhead, J., Smart, S. M., & Roy, D. B. (2019). The design, launch and assessment of a new volunteer-based plant monitoring scheme for the United Kingdom. PLoS ONE, 14(4). <https://doi.org/10.1371/journal.pone.0215891>

Rodwell, J. S. (1998). British Plant Communities. Cambridge University Press.

Stace, C. (2019). New Flora of the British Isles (4th ed.). Cambridge University Press.

# Appendix A


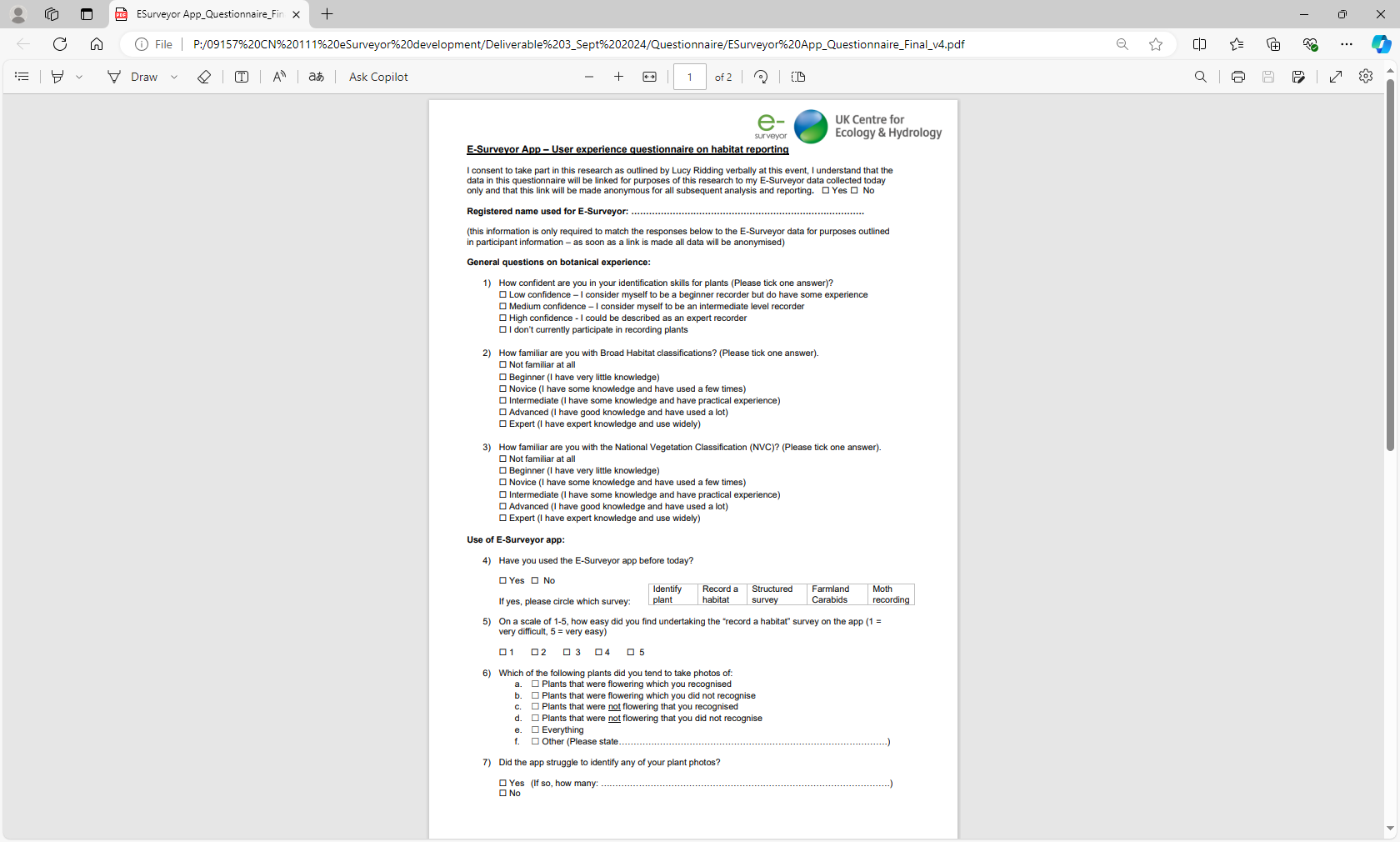


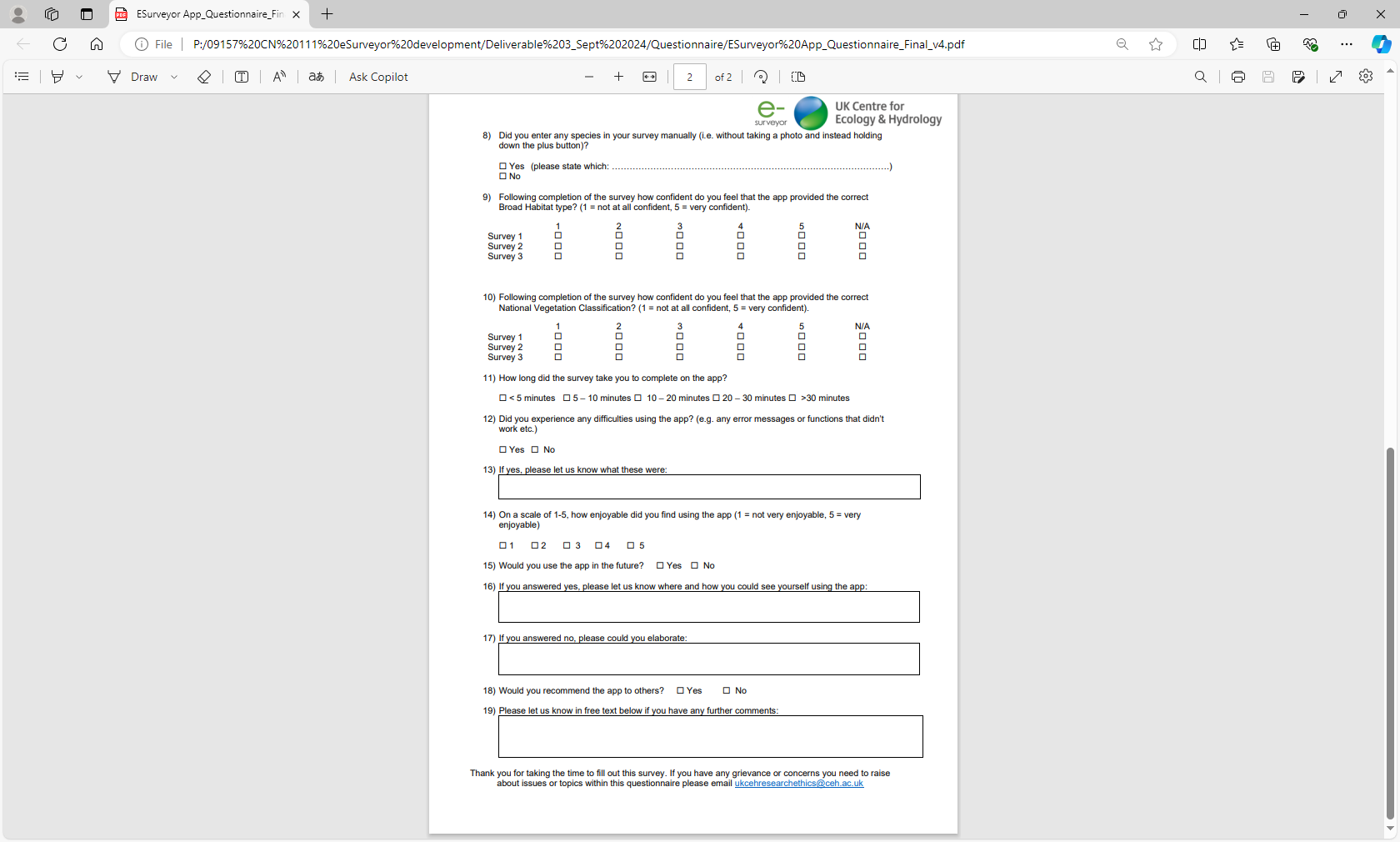

Supplement: Supplementary file 1 — Data S1: Supporting Information. [file ECE3-16-e73901-s002.docx]
