# Supplementary material for: Evaluation of the e‐Surveyor Mobile Application for Undertaking Plant Surveys and Predicting Habitat Type
Source: Ecol Evol. 2026 Jun 29;16(7):e73901. doi: 10.1002/ece3.73901 (PMC13314545; doi:10.1002/ece3.73901)
Supplement: Supplementary file 2 — Figure S1: Scatterplot showing the number of plant species recorded per 25 m2 plot by participants and experts for the three different habitat types during the e‐Surveyor workshops. Only a single participant's data is used per plot. Table S1: Parameter estimates and confidence intervals of generalised linear mixed models (poisson) comparing the numbers of species identified by user types (expert and participant) in broad habitat types (improved, neutral and calcareous grasslands) as additive terms. Model AIC = 893.20, marginal R 2 = 0.75, conditional R 2 = 0.82. Random effects plot std. dev. = 0.16. 46 plot groups. Table S2: Parameter estimates and confidence intervals of generalised linear mixed models (binomial) of the likelihood of species being correctly recorded on the e‐Surveyor application with plant type, flowering and abundance as interaction terms. Model AIC = 1334.44, marginal R 2 = 0.26, conditional R 2 = 0.50. Random effects species std. dev. = 1.10, app user std. dev. = 0.65. 139 species groups, 46 plot groups. Appendix A: Questionnaire completed by participants after completing an e‐Surveyor habitat survey at one of the workshops. Appendix B: DHARMa residual diagnostics: number of species. Appendix C: DHARMa residual diagnostics: species identification accuracy. Appendix D: DHARMa residual diagnostics: habitat accuracy. [file ECE3-16-e73901-s001.docx]

**Figure S1.** Scatterplot showing the number of plant species recorded per 25 m^2^ plot by participants and experts for the three different habitat types during the e-Surveyor workshops. Only a single participant’s data is used per plot.


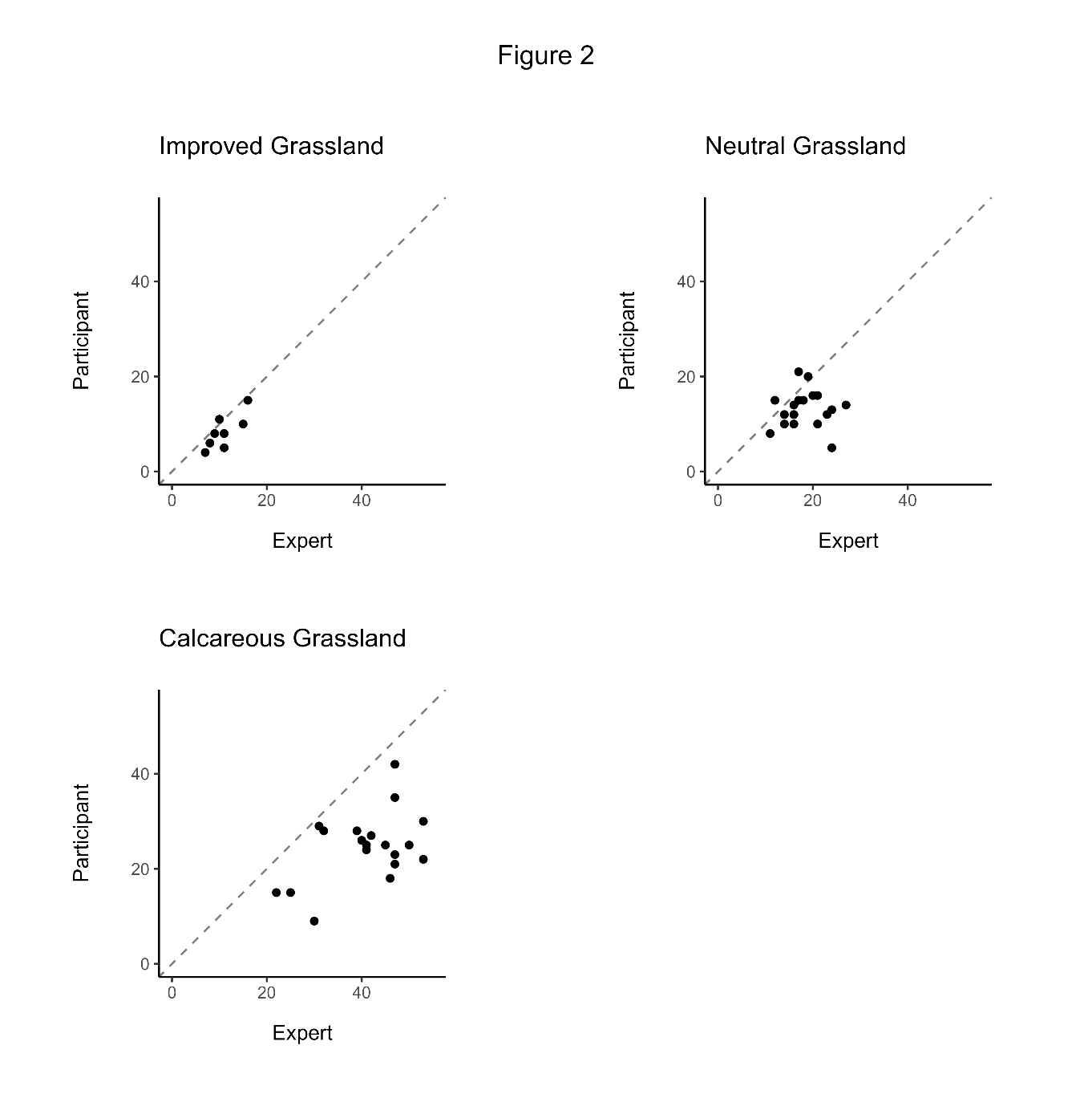


**Table S1.** Parameter estimates and confidence intervals of generalised linear mixed models (poisson) comparing the numbers of species identified by user types (expert and participant) in broad habitat types (improved, neutral and calcareous grasslands) as additive terms. Model AIC = 893.20, marginal *R*^2^ = 0.75, conditional *R*^2^ = 0.82. Random effects plot std. dev. = 0.16. 46 plot groups.

| Explanatory variable | Coefficient | SE | 2.5% CI | 97.5% CI | z value | p-value |
| --- | --- | --- | --- | --- | --- | --- |
| UserParticipant | -0.22 | 0.04 | -0.30 | -0.14 | -5.29 | <0.001 |
| HabitatNeutralgrassland | 0.52 | 0.10 | 0.32 | 0.71 | 5.23 | <0.001 |
| HabitatCalcareousgrassland | 1.20 | 0.09 | 1.02 | 1.39 | 12.72 | <0.001 |

**Table S2.** Parameter estimates and confidence intervals of generalised linear mixed models (binomial) of the likelihood of species being correctly recorded on the e-Surveyor application with plant type, flowering and abundance as interaction terms. Model AIC = 1334.44, marginal *R*^2^ = 0.26, conditional *R*^2^ = 0.50. Random effects species std. dev. = 1.10, app user std. dev. = 0.65. 139 species groups, 46 plot groups.

| Explanatory variable | Coefficient | SE | 2.5% CI | 97.5% CI | z value | p-value |
| --- | --- | --- | --- | --- | --- | --- |
| PlantTypeGrass/sedge | -2.35 | 0.77 | -3.85 | -0.84 | -3.06 | 0.002 |
| Flowering1 | 1.45 | 0.67 | 0.13 | 2.77 | 2.15 | 0.032 |
| AbundanceO | -1.11 | 0.55 | -2.18 | -0.03 | -2.02 | 0.043 |
| AbundanceR | -2.37 | 0.57 | -3.49 | -1.25 | -4.13 | <0.001 |
| PlantTypegrass/sedge * Flowering1 | 0.35 | 0.87 | -1.35 | 2.05 | 0.41 | 0.685 |
| PlantTypegrass/sedge * AbundanceO | -0.48 | 0.90 | -2.25 | 1.28 | -0.54 | 0.593 |
| PlantTypegrass/sedge * AbundanceR | 0.86 | 0.97 | -1.04 | 2.76 | 0.89 | 0.376 |
| Flowering1 * AbundanceO | -0.45 | 0.71 | -1.85 | 0.94 | -0.64 | 0.524 |
| Flowering1 * AbundanceR | -0.61 | 0.79 | -2.17 | 0.94 | -0.77 | 0.440 |
| PlantTypegrass/sedge * Flowering1 * AbundanceO | 0.79 | 1.04 | -1.25 | 2.83 | 0.76 | 0.448 |
| PlantTypegrass/sedge * Flowering1 * AbundanceR | -0.69 | 1.26 | -3.15 | 1.78 | -0.55 | 0.586 |

**Supporting information**

**Appendix A.** Questionnaire completed by participants after completing an e-Surveyor habitat survey at one of the workshops.


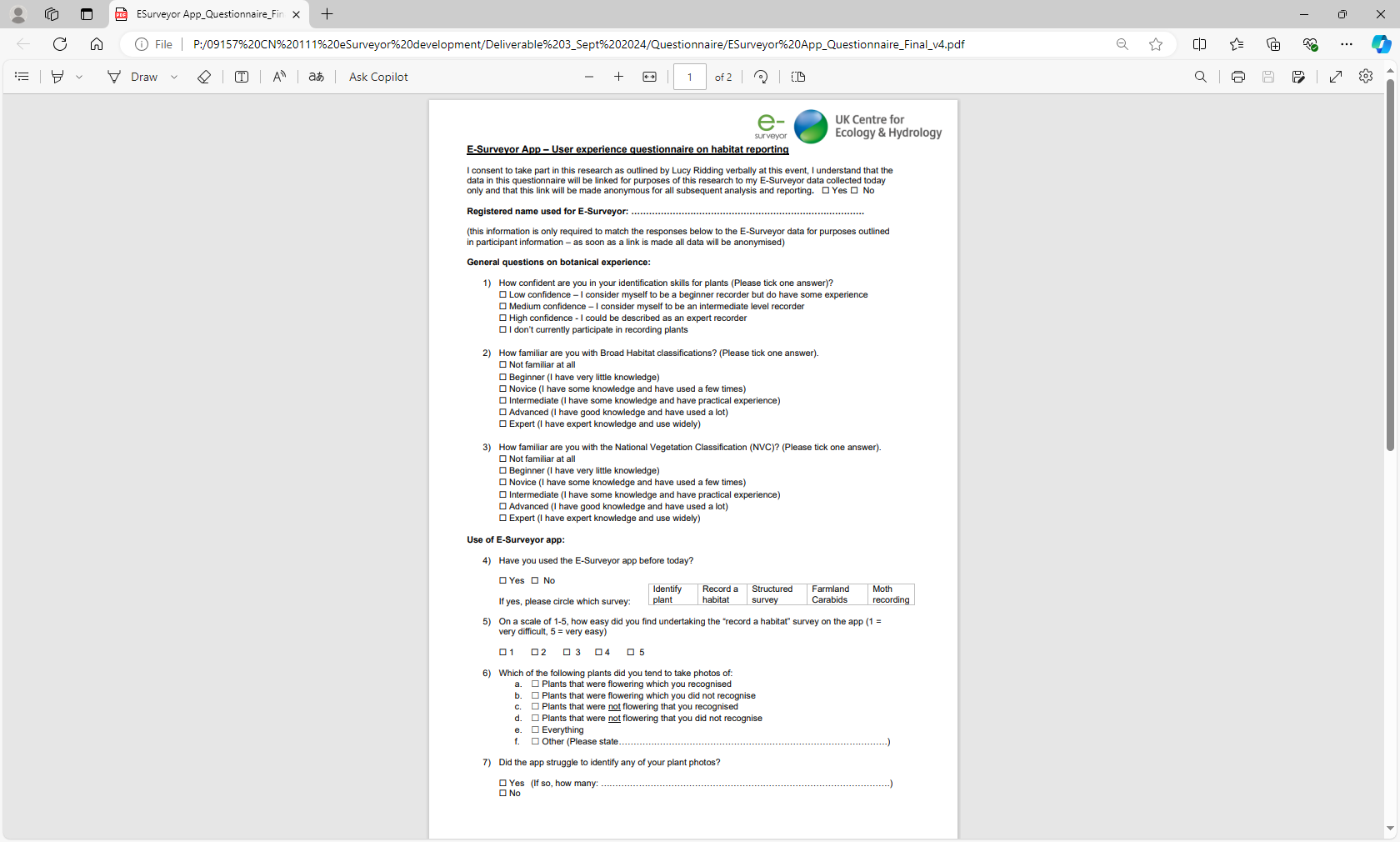


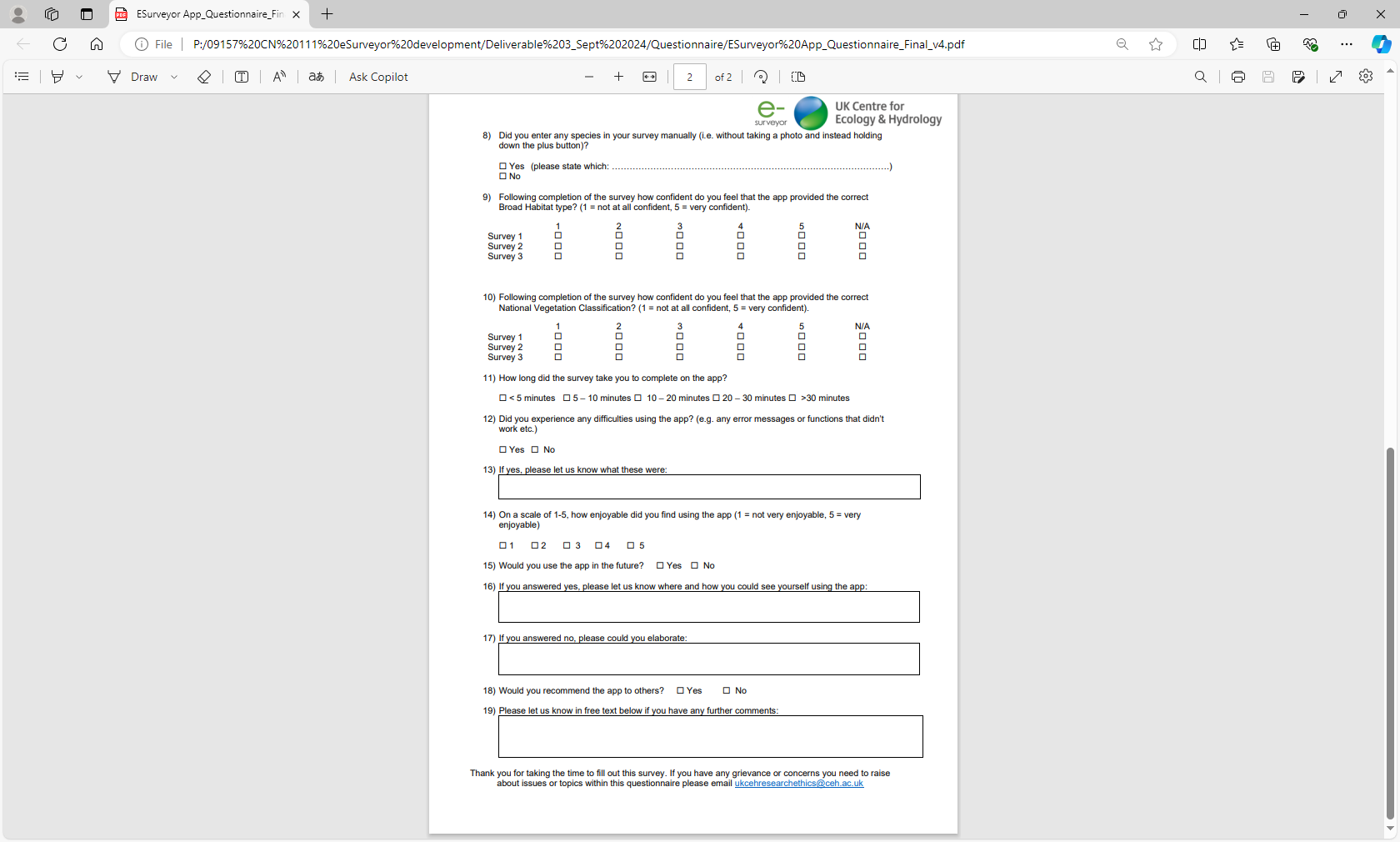


**Appendix B. DHARMa residual diagnostics: number of species.**

**B.1. User type and broad habitat**

Model: glmer(species_richness ~ user + broad_habitat +(1|unique_plot_id), family=poisson(), na.action = "na.fail", data=sr)


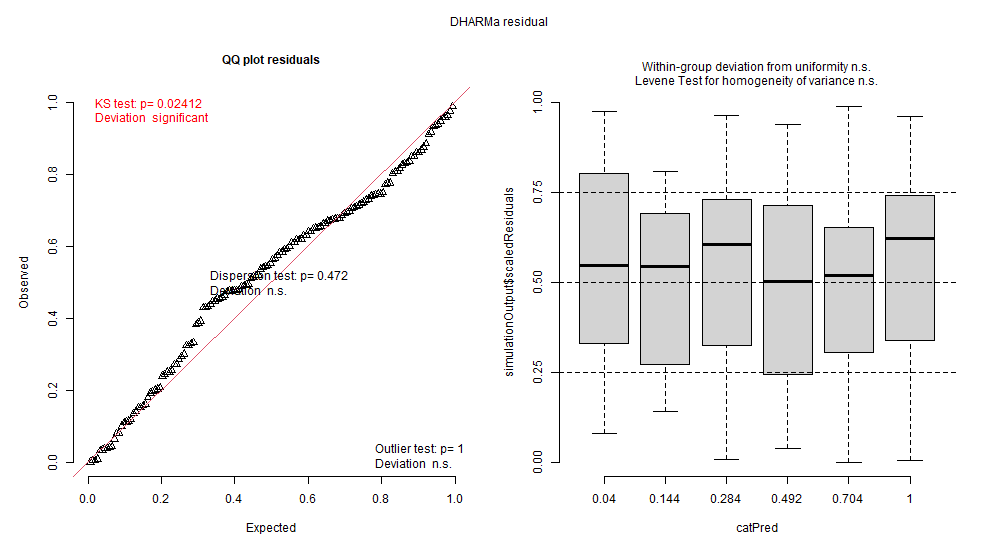


**Figure 1.** DHARMa residual plots showing KS, dispersion, outlier and Levene homogeneity of variance tests. We were not concerned about the significant KS test.


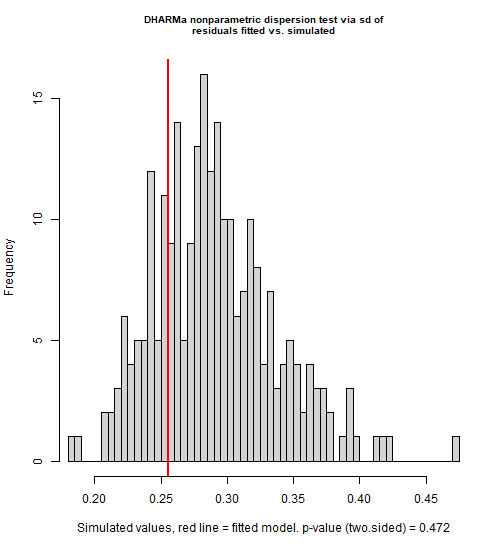


**Figure 2.** DHARMa nonparametric dispersion test via sd of residuals fitted vs. simulated.

Dispersion = 0.8805, p-value = 0.472, alternative hypothesis: two-sided.

**B.2. User confidence**

Model: glmer(species_richness ~ confidence + (1|id_num) + (1|broad_habitat), family=poisson(), na.action = "na.fail", data=sr_user)


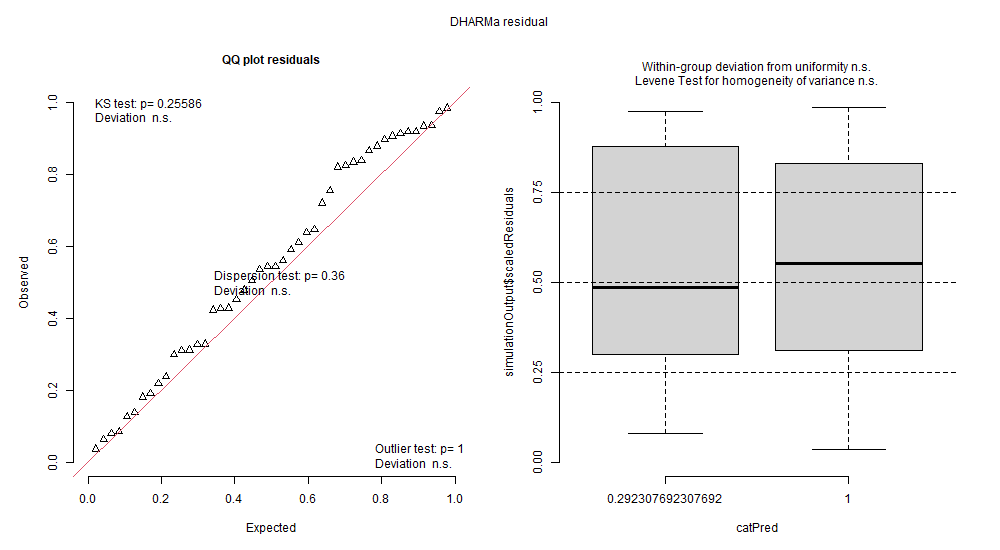


**Figure 3.** DHARMa residual plots showing KS, dispersion, outlier and Levene homogeneity of variance tests.


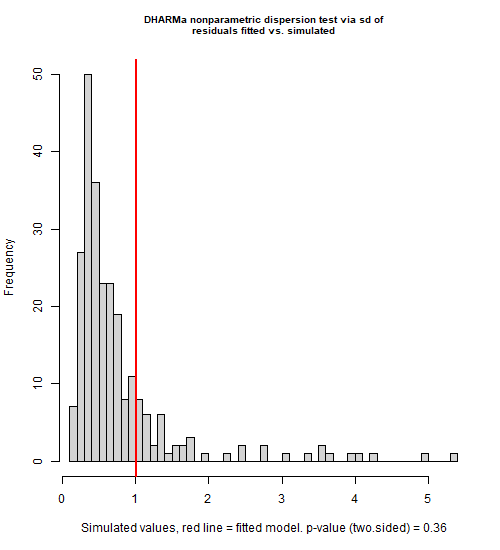


**Figure 4.** DHARMa nonparametric dispersion test via sd of residuals fitted vs. simulated. Dispersion = 1.3039, p-value = 0.36, alternative hypothesis: two-sided.

**Appendix C. DHARMa residual diagnostics: species identification accuracy.**

Model: glmer(as.numeric(status) ~ type * flowers * dafor_short * (1|app_user_id) + (1|Species), family=binomial(), data = species)


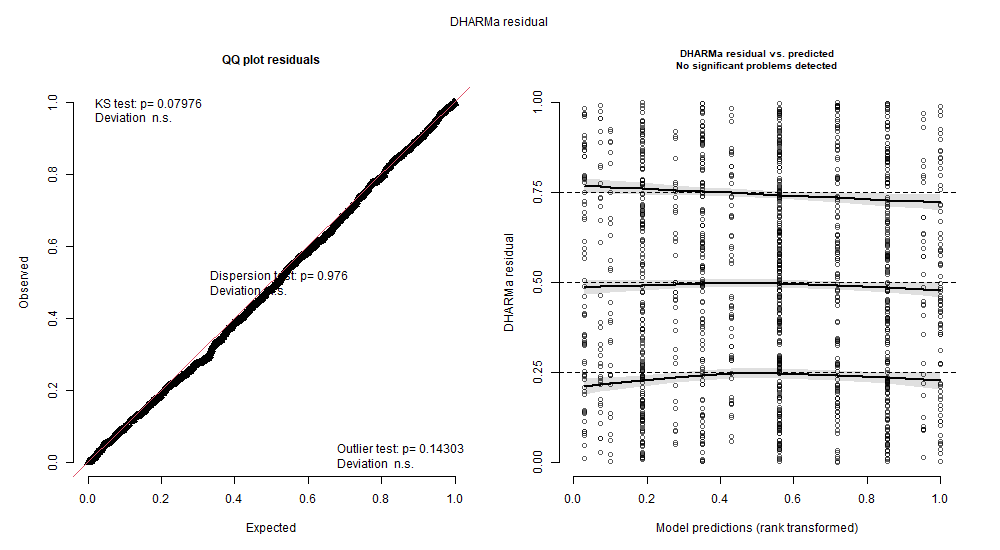


**Figure 1.** DHARMa residual plots showing KS, dispersion, outlier and Levene homogeneity of variance tests.


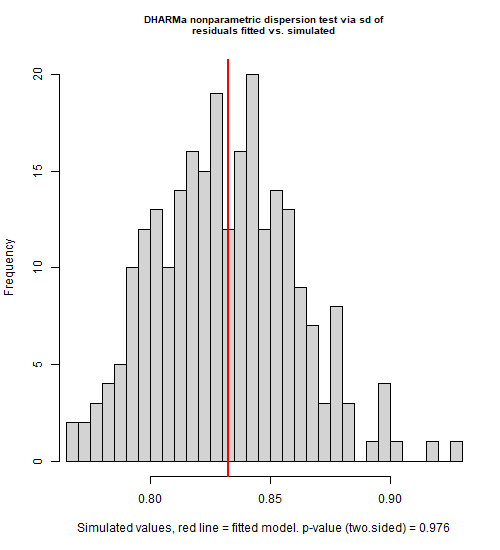


**Figure 2.** DHARMa nonparametric dispersion test via sd of residuals fitted vs. simulated. Dispersion = 1.0011, p-value = 0.976, alternative hypothesis: two-sided.

**Appendix D. DHARMa residual diagnostics: habitat accuracy.**

Model: glm(as.numeric(Correct_Top_BH) ~ scale.correct_sp_percent, family=binomial(), data = survey)


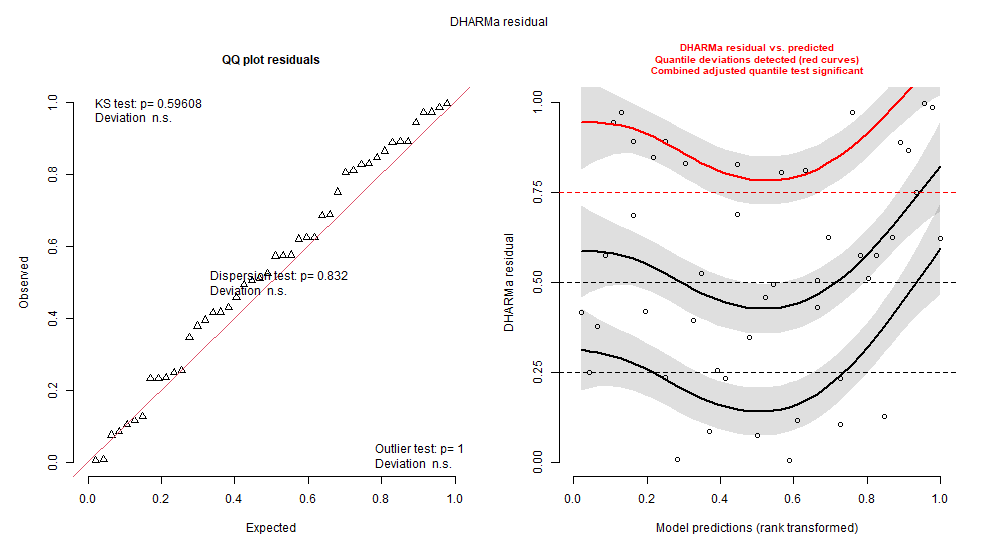


**Figure 1.** DHARMa residual plots showing KS, dispersion, outlier and Levene homogeneity of variance tests. qu = 0.25, log(sigma) = -2.673932 : outer Newton did not converge fully. We were not concerned about the significant combined adjusted quantile test.


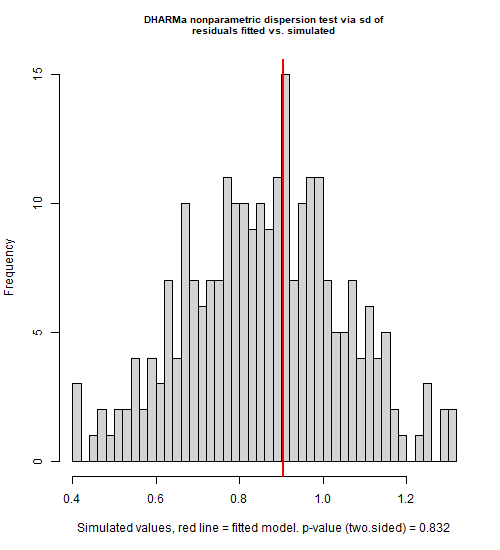


**Figure 2.** DHARMa nonparametric dispersion test via sd of residuals fitted vs. simulated. Dispersion = 1.0506, p-value = 0.832, alternative hypothesis: two-sided.
